# Genome-wide association study of seedling–plant resistance to stripe rust in bread wheat (*Triticum aestivum* L.) genotypes

**DOI:** 10.3389/fpls.2025.1554216

**Published:** 2025-05-02

**Authors:** Genet Atsbeha, Tilahun Mekonnen, Haftom Brhane, Mulugeta Kebede, Teklehaimanot Haileselassie, Kassahun Tesfaye

**Affiliations:** ^1^ Department of Applied Biology, Collage of Applied Natural Science, Adama Science and Technology University, Adama, Ethiopia; ^2^ Institute of Biotechnology, Addis Ababa University, Addis Ababa, Ethiopia; ^3^ Plant Breeding Department, Swedish University of Agricultural Sciences, Lomma, Sweden; ^4^ Department of Plant Biology and Biodiversity Management, Addis Ababa University, Addis Ababa, Ethiopia; ^5^ Bio and Emerging Technology Institute, Affiliated with Institute of Biotechnology, Addis Ababa University, Addis Ababa, Ethiopia

**Keywords:** genetic architecture, linkage disequilibrium, SNP markers, marker trait association, novel loci, *Puccinia striiformis*

## Abstract

Fungal diseases, such as stripe rust, are major bottlenecks in Ethiopian wheat production. They can significantly reduce yields and impact regional food security. To enhance Ethiopian wheat production, incorporating genetically resistant cultivars into breeding programs is essential. Accordingly, this study aimed at exploring the genome-wide association of seedling resistance in 178 wheat genotypes to identify genetic markers linked to yellow rust resistance. The panel was phenotyped for yellow rust seedling resistance at Kulumsa Agricultural Research Centre Pathology Laboratory. Additionally, the association panel was genotyped using the genotyping-by-sequencing (GBS) platform and a total of 6,788 polymorphic SNPs were used in genome-wide association analysis to identify effective yellow rust resistance genes. The Genome Association and Prediction Integrated Tool (GAPIT) was used to analyze marker–trait associations. The overall linkage disequilibrium (LD) decreased within an average physical distance of 31.44 Mbp at r^2^ = 0.2. Marker-trait association (MTA) analysis revealed 102 loci that are significantly (p = 0.001) related to yellow rust seedling–plant resistance. The majority of the discovered resistance quantitative trait loci (QTLs) were located on the same chromosomes as previously reported QTLs for yellow rust resistance, specifically on chromosomes 1A, 1B, 2A, 2B, 2D, 3A, 3B, 3D, 5A, 5B, 5D, 6A, 6B, 6D, 7A, 7B, and 7D. However, seven of the detected MTAs had not previously been documented in wheat literature or the International Wheat Genome Sequencing Consortium (IWGSC), suggesting that they may represent potentially novel loci for stripe rust resistance. Zooming in on QTL regions in the IWGSC RefSeq Annotation v1.1 revealed critical disease resistance-associated genes involved in plant defensive mechanisms against pathogen infections. The newly identified QTLs will be useful for marker-assisted wheat breeding to boost resistance to stripe rust.

## 1 Introduction

Wheat (*Triticum aestivum* L.) is one of the world’s major staple food crops, providing 21% of the total energy and 20% of the protein needs for approximately 4.5 billion people globally (Ramadas et al., 2019). In Ethiopia, bread wheat is a crucial staple crop, particularly in urban areas, contributing to nearly 15% of the daily calorie intake for a population of over 90 million (FAOSTAT, 2022). Wheat is also used as animal feed and for income generation (FAO, 2018). Furthermore, its importance extends beyond bread, biscuits, and pastry products but also for the production of starch and gluten (Hanson, 2022). In Ethiopia, wheat is one of the strategic food security crops (Bezabeh et al., 2015), ranking fourth after teff (*Eragrostis tef*), maize (*Zea* mays), and sorghum (*Sorghum bicolor*) in area coverage and third after maize and teff in total production (CSA, 2019). In 2021, approximately 1.95 million ha of land was covered with wheat, resulting in total national production of 5.2 million tons (FAOSTAT, 2022). The major wheat-growing areas in Ethiopia are located between 6 and 16° N and 35 and 42° E and at altitudes ranging from 1,500 to 3,000 m above sea level (masl). The most suitable areas for wheat production, however, fall between 1,900 and 2,700 masl. Furthermore, the central highlands as well as southeastern and northwestern parts of the country are Ethiopia’s main wheat-growing areas. In terms of regional contribution, the production of wheat originates from Oromia (57.4%) and more than 41% of the annual wheat production comes from only three areas in Oromia (Dubale, 2018). In 2020, Arsi, Bale and Shewa covered 75.5% of the total land used for wheat cultivation (Abboye, 2021). Irrespective of the significant increase in average wheat cultivation and production in Ethiopia, the national average of wheat productivity stands at 2.65 t ha^−1^, far lower than the global average of 3.6 t ha^−1^, resulting in a production deficit to meet the rising local demand (Gemechu and Tadese, 2018). Ethiopia satisfies 25%–35% of its domestic wheat demand through commercial imports and food aid (Agriculture Global Practice, 2018).

Fungal diseases such as yellow rust (stripe rust), stem rust, leaf rust, and septoria tritici blotch (STB) are the major bottlenecks for wheat production in Ethiopia. If not controlled, rust diseases can cause 50–100% yield loss (Chen, 2005) with an estimated cost of 5.5 billion USD per annum globally (Beddow et al., 2015). Among all rust diseases, yellow rust is the most significant threat to wheat production. It affects leaves, where the damage to photosynthetic tissues causes a reduction in the efficiency of light absorption and radiation (Bouvet et al., 2022). Furthermore, yellow rust limits yield by reducing the green leaf area, which supplies sugar to the developing seed. This is because flag leaves and second leaves are the most important for producing sugar for the developing grain (Murray et al., 2005). Since the flag leaf alone accounts for more than 70% of grain filling, its infection with stripe rust results in significant yield loss (Marsalis and Goldberg, 2006).

In Ethiopia, *P. striiformis* is the most common rust pathogen, causing significant wheat yield losses of up to 100% in the worst seasons (Tadesse et al., 2018). Yellow rust spores migrate quickly, can travel long distances, and produce diverse populations, which makes controlling the disease difficult (Vergara-Diaz et al., 2015). Crop yield loss due to stripe rust requires well-organized plant disease management and control (Sarrocco and Vannacci, 2018). However, the broad use of fungicides to treat fungal diseases has the potential to affect environment and animal welfare (Bhandari et al., 2019). These pollutants can contaminate water supplies and soil, endangering aquatic ecosystems and upsetting natural ecological balance. Meanwhile, the most cost-effective method for managing yellow rust is to produce resistant cultivars, which reduces the undesirable environmental and human health effects associated with fungicides use (Chen, 2005; Hansona et al., 2016). Varietal resistance, on the other hand, can be easily overcome due to the introduction of novel *P. striiformis* races or mutations (Wellings, 2011). Because the production of resistant cultivars is the most effective, economical, and environmentally safe management approach with limitless value for farmers (Chen, 2005), effective resistance breeding requires accurate race (isolate)-based studies, as well as the discovery and use of novel resistance genes that outperform the commonly virulent races (Admassu et al., 2012). Accordingly, this study aimed at conducting a genome-wide association study (GWAS) on seedling-stage resistance to stripe rust in 178 wheat genotypes to identify genetic markers associated with yellow rust resistance.

## 2 Methodology

### 2.1 Plant materials and evaluation of stripe rust resistance in seedling

The current study used 178 bread wheat germplasms, including 163 recombinant inbred lines obtained from the International Maize and Wheat Improvement Center (CIMMYT-Mexico) and 13 commercial cultivars cultivated in Ethiopia (**Supplementary Table 1**). The germplasms from CIMMYT comprised 6 genotype lines from the National Variety Trial, 5 from the Adaptation Trial, 34 from the High Rain Wheat Screening Nursery, 49 from the International Bread Wheat Screening Nursery, 54 from the International Septoria Observation Nursery, 14 from the High Rain Wheat Yield Trial, and 34 from the High Rain Wheat Screening Nursery, and the remaining three genotypes were from the Preliminary Variety Trial. In 2023, the bread wheat germplasms were tested in the pathology laboratory of the Kulumsa Agricultural Research Centre (KARC). The wheat genotype Kingbird (G40) was used as a standard check. The experiment was set up as an alpha-lattice design with two replications, and it was repeated three times.

The modified Cobb’s Scale (Peterson et al., 1948) was used to assess disease severity, with scores ranging from 0% to 90%. Additionally, the genotypes’ field reaction (FR) to stripe rust infection was graded using the approach of Roelfs et al. (1992): Immune = no uredinia or any macroscopic indication of infection; R: resistant with tiny uredinia surrounded by necrosis. MR indicates moderately resistant with medium- to large-sized uredinia surrounded by necrosis. MS = moderately resistant to moderately susceptible; MS = medium- to large-sized uredinia surrounded by chlorosis; S = susceptible, large-sized uredinia with no necrosis or chlorosis. When disease severity exceeded or equaled 50% and a susceptible reaction (S) was recorded on the spreader rows, the plant resistance response was scored, resulting in a combined value of 50S (Alemu et al., 2021).

### 2.2 Data analysis

#### 2.2.1 Genomic DNA extraction and genotyping by sequencing

For genomic DNA extraction, wheat seedlings were grown under greenhouse conditions at the National Agricultural Biotechnology Research Center (NABRC). Leaves from 2-week-old seedlings were collected and dried overnight at 50°C. The samples were delivered to the BecA-ILRI Hub laboratory in Nairobi, Kenya, for SNP genotyping using Diversity Arrays Technology sequencing (DArTseq™). Genomic DNA was isolated using the NucleoMag Plant Genomic DNA Extraction Kit (MACHEREY-NAGEL GmbH & Co. KG, Germany) according to the manufacturer’s instructions. DNA quality and quantity were assessed using a NanoDrop spectrophotometer and 1% agarose gel electrophoresis, respectively. SNP genotyping was performed using the genotype-by-sequencing (GBS) method on an Illumina HiSeq 2500, as described by Elshire et al. (2011).

#### 2.2.2 Quality control and SNP calling

The DArTSeq SNP markers were scored using the DArTsoft14 tool, which was integrated into the KDCompute plug-in system and aligned with the Chinese Spring Wheat RefSeq v1.0 reference genome (IWGSC, 2018). SilicoDArT and SNP markers were both rated binary (1/0), indicating whether marker data were present or absent in each sample’s genomic representation (Akbari et al., 2006). Marker quality was maintained by filtering or eliminating monomorphic markers, markers with poor call rates (>30% missing), and markers with minor allele frequencies (MAF < 5%). Genotypes with more than 30% missing marker data were also removed from the analysis.

#### 2.2.3 Population structure analysis

The population mixing pattern was determined using Bayesian model-based clustering in STRUCTURE version 2.3.4 (Pritchard et al., 2000). The STRUCTURE software was used with the admixture model, correlated allele frequencies, and a burn-in period of 50,000 and 100,000 Markov chain Monte Carlo (MCMC) replications for the hypothetical subpopulation K, from 1 to 10 with 10 iterations. The optimal K value was determined using STRUCTURE HARVESTER version 0.6.92, as stated by Evanno et al. (2005). The ideal K bar graph was calculated using the CLUMPAK beta version (Kopelman et al., 2015). To determine the population geographical distribution and clustering, principal component analysis (PCA) was performed using the Genome Association and Prediction Integrated Tools (GAPIT) software.

#### 2.2.4 Genome-wide association analysis

The marker-trait association analysis was carried out using GAPIT software (Lipka et al., 2012). GWAS was performed on three yellow rust disease variables, namely, disease severity, plant response, and infection coefficient. The marker-trait association study included 6,788 robust SNPs with call rates >70% and MAF >5%. Missing SNPs were imputed using Optimum Impute ver. 1.0.0, which is based on the KNN imputation algorithm in the KDcompute_plugin system. The LD measure R^2^ ver.0.2.2 in the KDcompute_plugin system was used to calculate the marker distribution on each chromosome. In addition, TASSEL Ver. 5 (Bradbury et al., 2007) was used to compute LD evaluations (r^2^ and p-value) for the A and B and sub-gnomes, as well as the whole genome. The LD decay curve was drawn at cutoff r^2^ = 0.1 to allow an easy comparison with various previous studies in wheat.

The Iteratively Nested Keyway (BLINK) model, which is included in the GAPIT R package (Tang et al., 2016), was used to conduct GWAS. BLINK was chosen for its great computational efficiency and statistical power in controlling false associations caused by population structure and kinship. Additionally, Van-Raden (2008) approach was used to compute the kinship (K) matrix. The model’s fitness to manage population structure and family relatedness of the research samples was evaluated using a quantile–quantile (QQ) plot generated using −log10 p-values. Marker-trait associations (MTA) were regarded as significant if they exceeded the nominal p-values of 0.001 or −log10 (p-values) = 3. To identify relevant MTAs, Manhattan plots and Q–Q plots were used in conjunction with the R package qqman (Turner, 2014). The identified significant MTAs were grouped into QTLs based on chromosomal LD decay. Candidate genes in important regions were annotated using MTAs from the recently released IWGSC RefSeq Annotation v1.1, which is found at https://wheat-urgi.versailles.inrae.fr/SeqRepository/Annotations. To find the candidate genes, ± 4 Mb from the QTLs’ physical position was used. KnetMiner (http://knetminer.rothamsted.ac.uk) was used to explore details on genes associated with stripe rust resistance, helping to identify key genes involved in the plant’s defense mechanisms.

## 3 Results

### 3.1 SNP data

**Figure 1:**
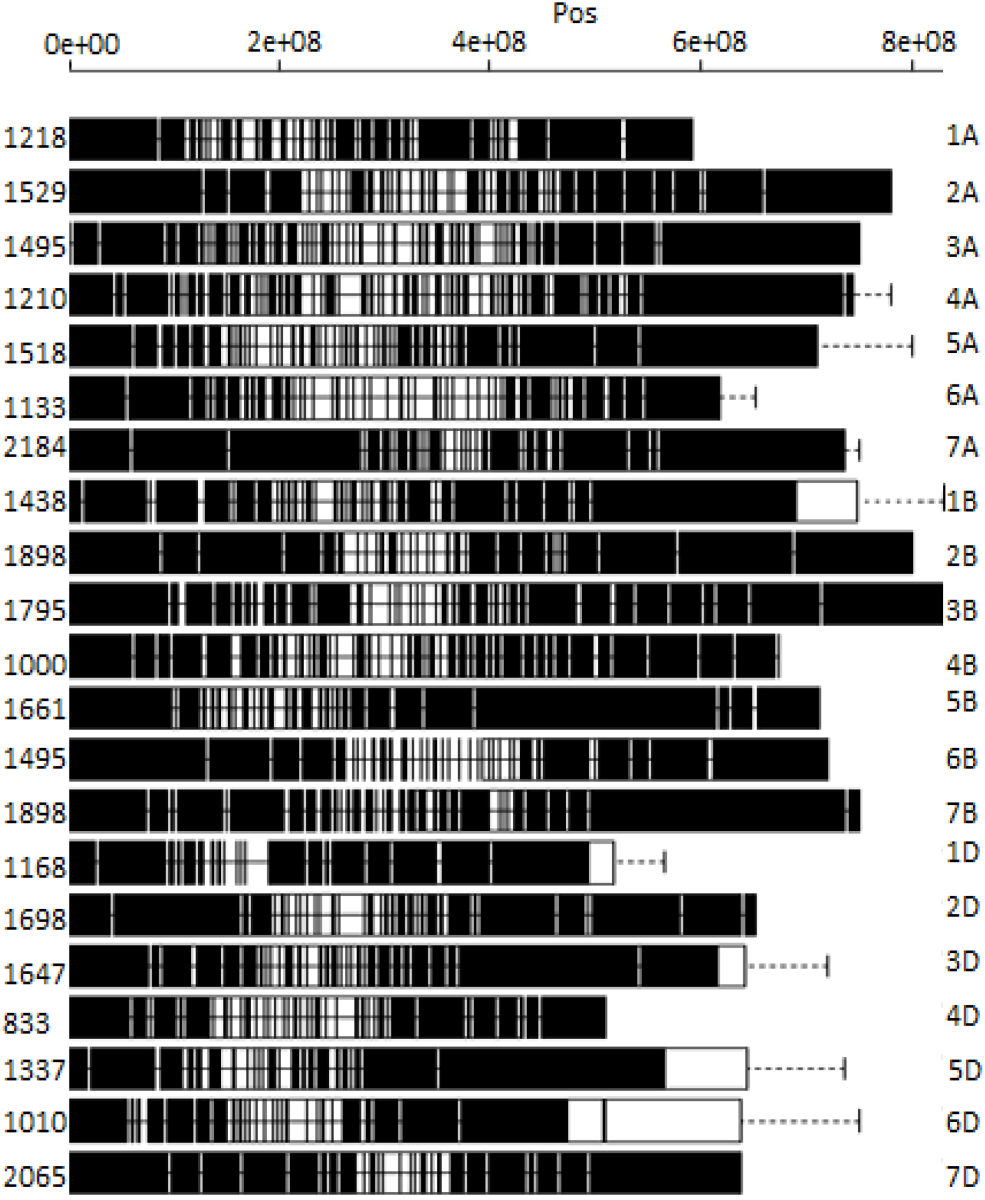
Distribution of DArTSeq SNPs on 21 bread wheat chromosomes.

DArTSeq genotyping of 178 bread wheat germplasms yielded a total of 35,672 SNPs (**Figure 1**). The three wheat sub-genomes (A, B, and D) had 10,317, 10,979, and 9,756 SNPs, respectively (**Figure 1**). Among the 21 wheat chromosomes, chromosome 4D had the fewest SNPs (833), whereas chromosome 7D had the highest (2,065). A total of 6,788 SNP markers were used following filtering with call rates >70% and minor allele frequencies >0.05. SNPs were distributed as 2,410 SNPs on the A sub-genome and 2,872 on the B sub-genome, and the filtered 6,788 SNPs were used in the current study.

### 3.2 Population structure analysis

**Figure 2:**
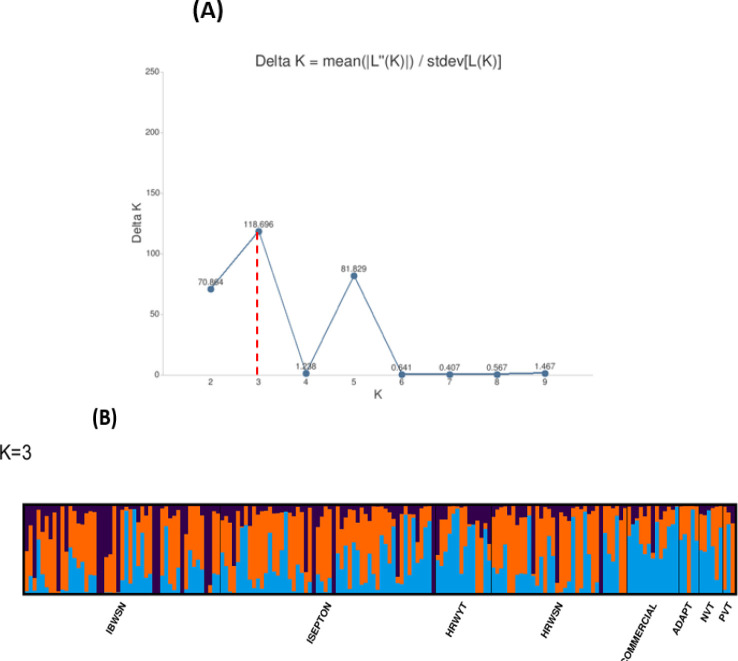
Population structures of 178 bread wheat genotypes representing eight populations. **(A)** Best delta K value estimated, and the pick at k = 3 indicates the number of subpopulations in wheat panel. **(B)** Estimated population structure for K = 3 according to the breeding materials. The different (blue orange and black) colors represent genetic groups or subpopulations: the x-axis represents individual samples, and the y-axis represents the proportion of ancestry to each cluster. Population abbreviations: IBWSN, International Bread Wheat Screening Nursery; ISEPTON, International Septoria Observation Nursery; HRWYT, High Rain Wheat Yield Trial; HRWSN, High Rain Wheat Screening Nursery; ADAPT, Adaptation trial; NVT, National Verification Trial; PVT, Preliminary Verification trial.

**Figure 3:**
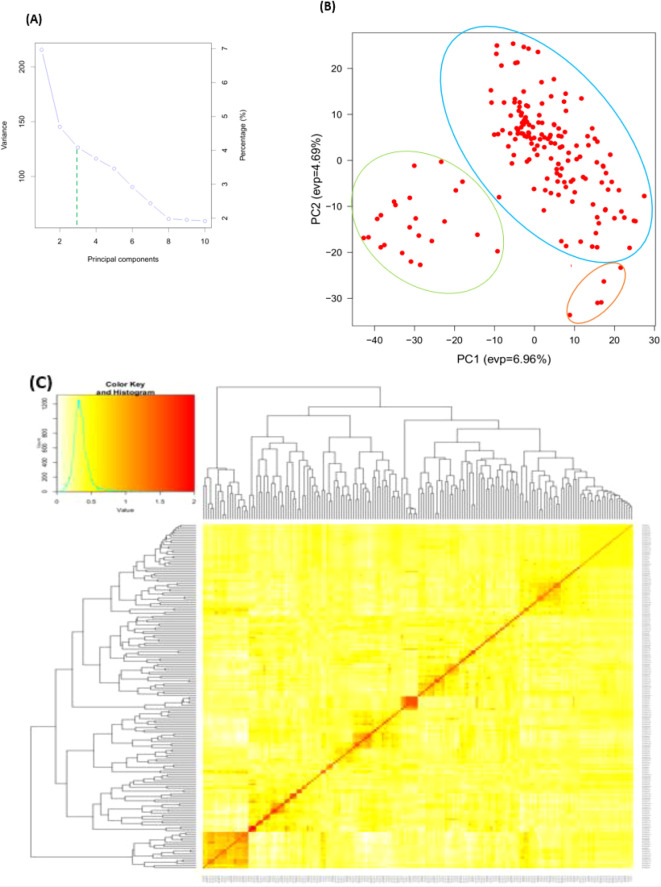
Principal component and familiar relatedness analysis of 178 wheat genotypes using 6,788 SNP markers. **(A)** Scatter plot and **(B)** 3D plots of the principal components. **(C)** Kinship displayed through heat map and a tree out of the heat map. The kinship values showed a normal distribution (turquoise curve), and orange represents weak correlation between pairs of individuals in the panel whereas red shows high correlation. The resulted clustering tree is indicated outside of the matrix.

Three subpopulations were inferred from the STRUCTURE program output (**Figure 2A**). The three clusters (**Figure 2B**) demonstrated a high degree of genetic mixing, indicating that the wheat populations in the study are closely related. All of the individual genotypes inherited genes from all three subpopulations. The presence of three clusters in the association with higher admixture was also supported by the scatter plot (**Figure 3A**) and the 2D plot of the first three principal components (**Figure 3B**), where the association panel’s fluctuation was primarily explained by the first two PCs’ (PC1 and PC2) coordinates. In marker-trait association studies, it is crucial to include both population structure (Q) and kinship (K) as covariates. Kinship analysis also validated the existence of cryptic familial relatedness (**Figure 3C**).

### 3.3 Linkage disequilibrium analysis

**Figure 4:**
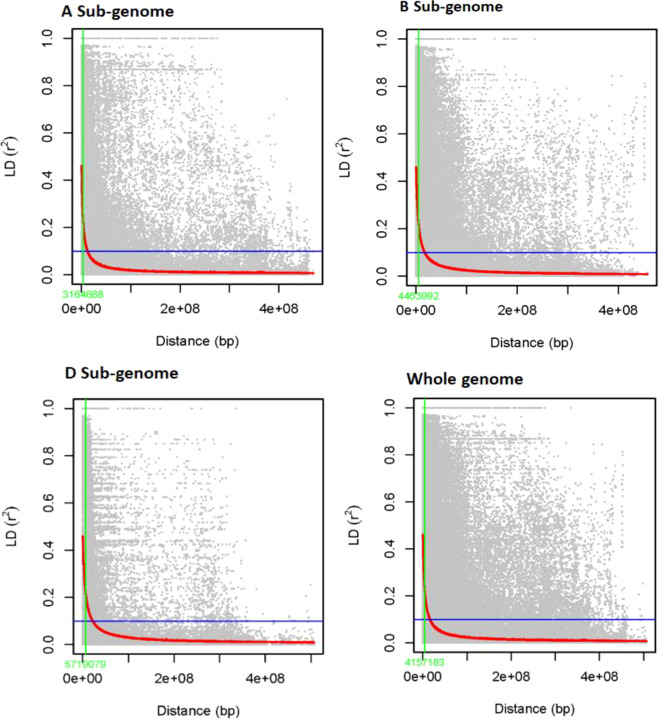
Sub-genome A, sub-genome B, sub-genome D, and genome-wide LD decay plots over physical distance based on 6,788 SNP markers. The red curve represents the model fits to LD decay. The vertical green line indicates the intersection between the critical r^2^ value and the average map distance to determine QTL confidence intervals.

The LD was estimated for 6,788 SNP markers distributed among 178 genotypes. Allele LDs differ between chromosomes and sub-genomes (**Supplementary Table 2**). Overall, 97,723 (27.61%) of the 338,125 marker pairings with average LD values of r^²^ = 0.11 revealed significant LD (p < 0.01; see **Figure 4**). The B sub-genome has the most marker pairs (143,600, or 42.47%), whereas the D sub-genome has the fewest marker pairs (75,300, or 22.27%). SNPs on the B sub-genome had the highest LD, with a mean r^²^ value of 0.1187. The LD between SNPs decreased across all chromosomes at the LD cutoff r^²^ = 0.2 over a physical distance of 31.44 Mbp. Marker pairings on chromosomes 4D and 2D had the lowest (r^²^ = 0.03) and highest (r^²^ = 0.21) correlations (**Supplementary Table 2**). The LD decay at cutoff r^²^ = 0.1 was observed at ~4.2 Mb in the complete panel, whereas it was observed at ~3.2, 4.5, and 5.7Mb in A, B, and D genomes, respectively. The highest LD decay was observed in the D genome (5.7 Mb), followed by the B (4.5 Mb) and B (3.2 Mb) genomes.

### 3.4 Genome-wide association study

The marker-trait association (MTA) methodology relied on linkage disequilibrium and Bayesian information. The BLINK statistical model-based association study revealed 102 SNPs that were significantly associated with yellow rust resistance at the seedling stage at a nominal p-value of 0.001, or −log10 (0.001) = 3. **Supplementary Table 3** lists the MTAs that exceeded the nominal p-value of 0.001 or −log10 (0.001) = 3 significant criteria for yellow rust resistance in terms of disease resistance, seedling response, and coefficient of infection. The allele identity, marker position, p-values, additive effects, and r² for the detected MTAs were also calculated. Among 102 identified MTAs, 31 (30.39%) MTAs conferred yellow rust resistance of disease severity, 41 (40.20%) for seedling resistance, and 30 (29.41%) for coefficient of infection.

Genome-wide scans for yellow rust resistance for each isolate identified considerable markers associated with yellow rust resistance for different isolates. In addition, MTA analysis for the Dekaa isolate identified 19 MTAs significantly associated with yellow rust for disease severity on chromosomes 2A, 2D, 3A, 6A, and 7A; 2 MTAs for seedling response on chromosomes 3B and 7A; and 8 significant MTAs conferring yellow rust resistance for coefficient of infection on chromosomes 2A, 2B, 2D, 3B, and 6A. Likewise, a GWA scan for yellow rust resistance for Hdasee isolate identified 25 MTAs, three of which were significantly associated with yellow rust for disease severity on chromosomes 1B, 3B, and 7D; 16 MTAs for seedling response on chromosomes 2A, 2B, 2D, 3A, 3D, 5A, 5D, 6D, and 7A; and six significant MTAs conferring yellow rust resistance for coefficient of infection on chromosomes 1A, 1B, 3D, 5D, 7A, and 7B.

**Figure 5:**
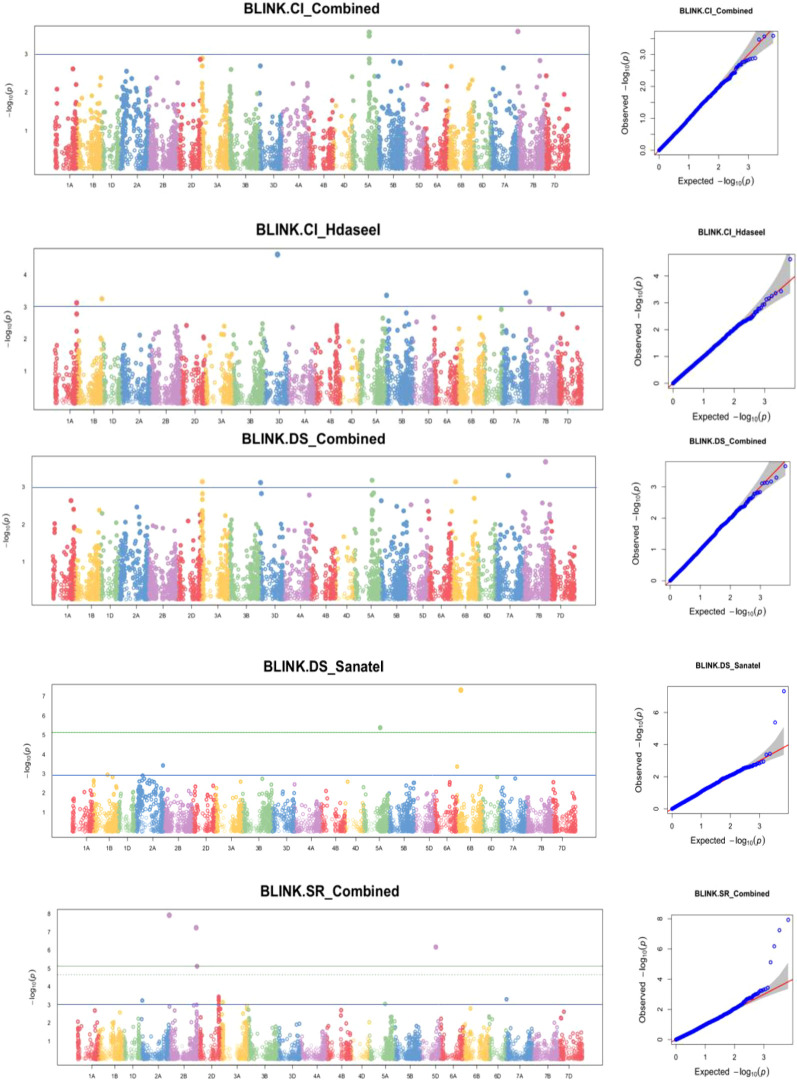
Some examples of Manhattan plots for yellow rust disease resistance traits. GWAS scans resulting with significant associations. Each dot represents an SNP. On the x-axis is the genomic position of the SNPs on the corresponding chromosomes indicated in different colors. On the y-axis is the *−log10* of the *P-value* depicting the significance of the association test. The horizontal orange line is the nominal *p* value 0.001 significance threshold used in the association studies of DS_DekaaI = disease severity for Dekaa isolate, DS_HdaseeI = disease severity for Hdasee isolate, DS_MeraroI = disease severity for Meraro isolate, DS_SanateI = disease severity for Sanate isolate, DS_Combined = disease severity combined. SR_DekaaI = seedling response for Dekaa isolate, SR_HdaseeI = seedling response for Hdasee isolate, SR_MeraroI = seedling response for Meraro isolate, SR_SanateI = seedling response for Sanate isolate, SR_Combined = seedling response combined, CI_DekaaI = confident of infection for the Dekaa isolate, CI_HdaseeI = confident of infection for the Hdasee isolate, CI_MeraroI = confident of infection for Meraro isolate, CI_SanateI = confident of infection for Sanate isolate, CI_Combined = confident of infection combined. The quantile–quantile (Q–Q) plots at the right side of the Manhattan plots indicate how well the used BLINK model accounted for population structure and kinship for each of the disease traits. In each plot, the observed *–log (P values)* from the fitted GWAS models (y-axis) are compared with their expected value (x-axis) under the null hypothesis of no association with the trait. Each blue dot represents a single nucleotide polymorphism; the red line is the model for no association.

Moreover, a GWA scan for the yellow rust resistance analysis for Meraro isolate revealed 27 MTAs that confer resistance to yellow rust. The analysis identified nine MTAs significantly associated with yellow rust for disease severity on chromosomes 2A, 3A, 7B, and 7D; 10 MTAs on chromosomes 2B, 3A, 3B, 7B, and 7D; and eight significant MTAs conferring yellow rust resistance for coefficient of infection on chromosomes 2A, 3A, 7B, and 7D. Likewise, a GWA scan for yellow rust resistance for Sanate isolate identified 22 MTAs, 4 of which were significantly associated with yellow rust disease severity on chromosomes 2A, 5A, and 6B; 13 MTAs for seedling response on chromosomes 2A, 2B, 2D, 3A, 5A, 5D, and 7A; and 5 MTAs for coefficient of infection on chromosomes 2A, 5A, and 7B. The combined measure of yellow rust resistance across traits for disease severity, seedling response, and coefficient of infection provided significant associations. It found 22 MTAs, including six for disease severity on chromosomes 3A, 3D, 5A, 6B, 7A, and 7B; 13 for seedling response on chromosomes 2A, 2B, 2D, 3A, 5A, 5D, and 7A; and three for coefficient of infection on chromosomes 5A and 7B (**Supplementary Table 3**, **Figure 5**, **Supplementary Figure 1**).

**Figure 6:**
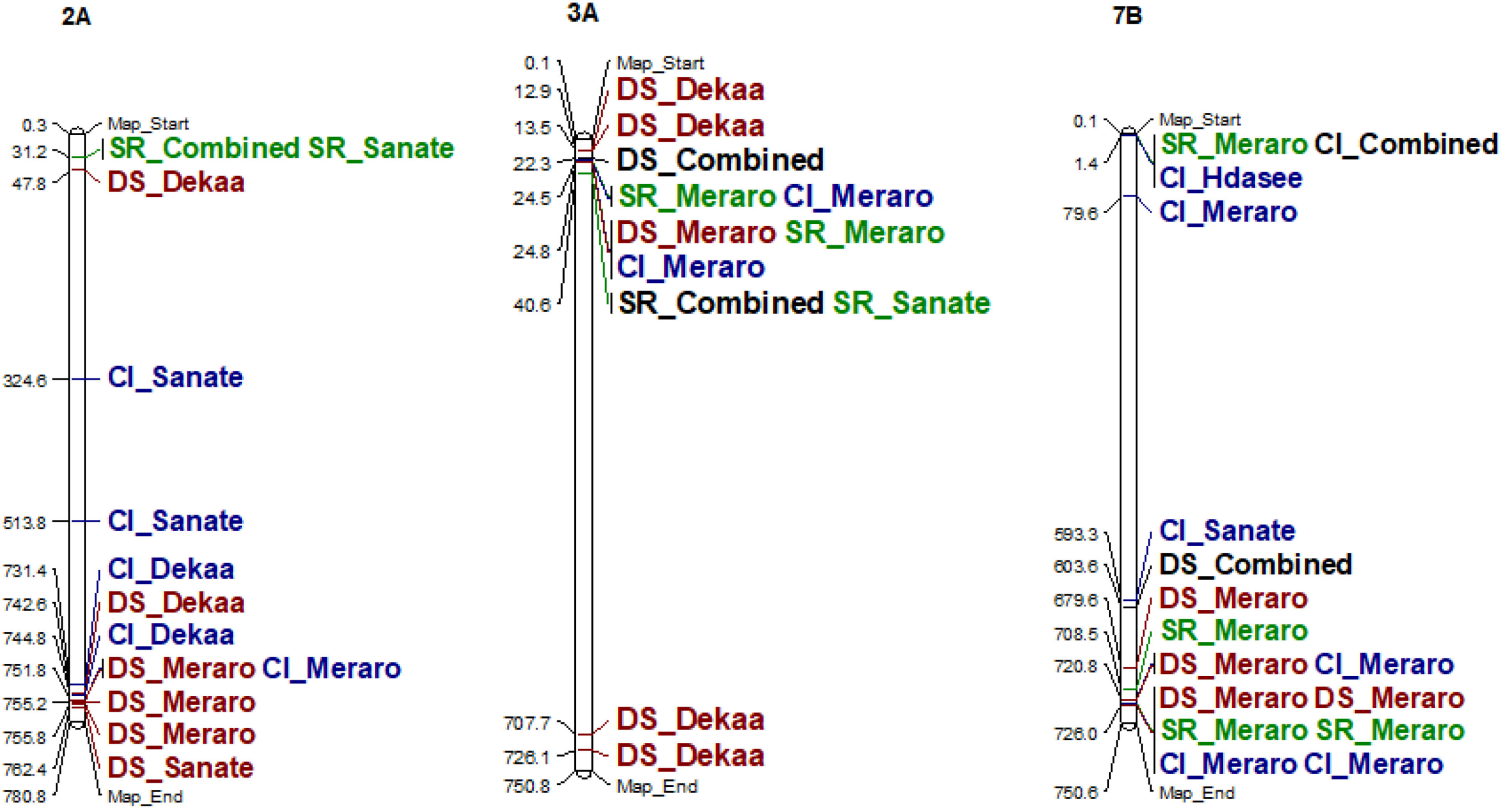
Some examples of genomic positions of detected putative QTLs effective for yellow rust resistance. Significant DArTSeq SNPs are presented according to their physical positions on chromosomes in millions base pairs. The putative QTLs identified in this study for the MTAs are indicated on the right sides of the bars. DS_DekaaI, disease severity for the Dekaa isolate; DS_HdaseeI, disease severity for Hdasee isolate; DS_MeraroI, disease severity for the Meraro isolate; DS_SanateI, disease severity for the Sanate isolate; DS_Combined, disease severity combined; SR_DekaaI, seedling response for the Dekaa isolate; SR_HdaseeI, seedling response for the Hdasee isolate; SR_MeraroI, seedling response for the Meraro isolate; SR_SanateI, seedling response for the Sanate isolate; SR_Combined, seedling response combined; CI_DekaaI, confident of infection for the Dekaa isolate; CI_HdaseeI, confident of infection for the Hdasee isolate; CI_MeraroI, confident of infection for the Meraro isolate; CI_SanateI, confident of infection for the Sanate isolate; CI_Combined, confident of infection combined.

Yellow rust resistance QTLs were identified by grouping the marker-trait associations (MTAs) based on their physical distance. MTAs on the same linkage group within the physical distance for LD decay specific for that chromosome were assigned to the same putative QTL if they fell within the 4.2-Mb interval based on the average whole genome LD decay (**Supplementary Table 4**, **Figure 6**, **Supplementary Figure 2**). Accordingly, the 102 MTA markers were assigned to 44 putative QTLs based on LD criteria. Therefore, the association analysis for yellow rust resistance for individual isolates found 25 putative QTLs. It was observed that some of the detected putative QTLs were effective for yellow rust resistance for more than two isolates. For instance, qYrSe.04, qYrSe.07, qYrSe.11, and qYrSe.12 contributed for stable yellow rust resistance for more than two isolates (**Supplementary Table 4**). Totally, 16 QTLs (qYrSe.04, qYrSe.08, qYrSe.11, qYrSe.12, qYrSe.13, qYrSe.17, qYrSe.18, qYrSe.24, qYrSe.26, qYrSe.30, qYrSe.33, qYrSe.35, qYrSe.36, qYrSe.40, and qYrSe.41) were stable across all isolates (**Table 1**).

**Table 1:** Putative QTLs stable across all isolates identified through bread wheat chromosomes for yellow rust resistance.

No	QTL	Chr	Position (bp)	Trait _Isolate
1	qYrSe.04	2A	31202549-47826068	SR_Combined, DS_Dekaa and CI_Sanate
2	qYrSe.07	2A	742581040-762415720	DS_Dekaa, DS_Meraro, DS_Sanate, CI_Dekaa and CI_Meraro
3	qYrSe.08	2B	1329109	SR_Combined and SR_Sanate
4	qYrSe.11	2B	742083622-750002860	SR_Combined, CI_Dekaa and SR_Sanate
5	qYrSe.12	2B	765297108-773807439	SR_Sanate and SR_Combined
6	qYrSe.13	2D	575366522-575705518	SR_Sanate and SR_Combined
7	qYrSe.17	3A	12868494-24780802	DS_Dekaa, DS_Meraro, SR_Meraro, CI_Meraro and DS_Combined
8	qYrSe.18	3A	40556131	SR_Sanate and SR_Combined
9	qYrSe.24	3D	9292491	DS_Combined
10	qYrSe.26	5A	437391883-471723485	DS_Sanate, SR_Sanate, DS_Combined, CI_Sanate, CI_Combined and SR_Combined
11	qYrSe.30	5D	425470918	SR_Combined
12	qYrSe.33	6B	113768715	DS_Sanate and DS_Combined
13	qYrSe.35	7A	13928165	DS_Sanate and SR_Combined
14	qYrSe.36	7A	339749362	SR_Meraro, CI_Hdasee and DS_Combined
15	qYrSe.40	7B	1440674	CI_Combined
16	qYrSe.41	7B	593285120 -603585766	CI_Sanate and DS_Combined

QTL, quantitative trait locus; Chr, chromosomes; Trait _Isolate, yellow rust resistance traits measured across isolates (Dekaa, Hdasee, Meraro, and Sanate); DS_DekaaI, disease severity for the Dekaa isolate; DS_HdaseeI, disease severity for the Hdasee isolate; DS_MeraroI, disease severity for the Meraro isolate; DS_SanateI, disease severity for the Sanate isolate; DS_Combined, disease severity combined; SR_DekaaI, seedling response for the Dekaa isolate; SR_HdaseeI, seedling response for the Hdasee isolate; SR_MeraroI, seedling response for the Meraro isolate; SR_SanateI, seedling response for the Sanate isolate; SR_Combined, seedling response combined; CI_DekaaI, confident of infection for the Dekaa isolate; CI_HdaseeI, confident of infection for the Hdasee isolate; CI_MeraroI, confident of infection for the Meraro isolate; CI_SanateI, confident of infection for the Sanate isolate; CI_Combined, confident of infection combined.

Moreover, the association analysis for yellow rust resistance for disease severity found that 20 putative QTLs were on chromosomes 1A (qYrSe.01), 2A (qYrSe.04 and qYrSe.07), 2D (qYrSe.16), 3A (qYrSe.17 and qYrSe.19), 3A (qYrSe.20), 3B (qYrSe.21), 3D (qYrSe.24 and qYrSe.25), 5A (qYrSe.26), 6A (qYrSe.31), 6B (qYrSe.32 and qYrSe.33), 7A (qYrSe.36 and qYrSe.38), 7B (qYrSe.41, qYrSe.42, and qYrSe.43), and 7D (qYrSe.44). It was observed that some of the detected putative QTLs were effective to yellow rust resistance for two or three traits, whereas others contributed to yellow rust resistance for disease severity only. For instance, qYrSe.38 contributed to yellow rust resistance for disease severity only. On the other hand, qYrSe.26 contributed to stable yellow rust resistance for all traits. Likewise, the association analysis for yellow rust resistance for seedling response and coefficient of infection found 21 and 19 putative QTLs, respectively (**Supplementary Table 4**). A total of eight QTLs (qYrSe.04, qYrSe.07, qYrSe.11, qYrSe.17, qYrSe.26, qYrSe.40, qYrSe.43, qYrSe.44) were stable across all traits (**Table 2**).

**Table 2:** Putative QTLs stable across all traits identified through bread wheat chromosomes for yellow rust resistance.

No	QTL	Chr	Position (bp)	Trait _Isolate
1	qYrSe.04	2A	31202549-47826068	SR_Combined, DS_Dekaa and CI_Sanate
2	qYrSe.07	2A	742581040-762415720	DS_Dekaa, DS_Meraro, DS_Sanate, CI_Dekaa and CI_Meraro
3	qYrSe.11	2B	742083622-750002860	SR_Combined, CI_Dekaa and SR_Sanate
4	qYrSe.17	3A	12868494-24780802	DS_Dekaa, DS_Meraro, SR_Meraro, CI_Meraro and DS_Combined
5	qYrSe.26	5A	437391883-471723485	DS_Sanate, SR_Sanate, DS_Combined, CI_Sanate, CI_Combined and SR_Combined
6	qYrSe.40	7B	1440674	SR_Meraro, CI_Hdasee and CI_Combined
7	qYrSe.43	7B	708445754-726016960	DS_Meraro, CI_Meraro and SR_Meraro
8	qYrSe.44	7D	3769072	DS_Meraro, CI_Meraro and SR_Meraro

QTL, quantitative trait locus; Chr, chromosomes; Trait _Isolate, yellow rust resistance traits measured across isolates (Dekaa, Hdasee, Meraro, and Sanate); DS_DekaaI, disease severity for the Dekaa isolate; DS_HdaseeI, disease severity for the Hdasee isolate; DS_MeraroI, disease severity for the Meraro isolate; DS_SanateI, disease severity for the Sanate isolate; DS_Combined, disease severity combined; SR_DekaaI, seedling response for the Dekaa isolate; SR_HdaseeI, seedling response for the Hdasee isolate; SR_MeraroI, seedling response for the Meraro isolate; SR_SanateI, seedling response for the Sanate isolate; SR_Combined, seedling response combined; CI_DekaaI, confident of infection for the Dekaa isolate; CI_HdaseeI, confident of infection for the Hdasee isolate; CI_MeraroI, confident of infection for the Meraro isolate; CI_SanateI, confident of infection for the Sanate isolate; CI_Combined, confident of infection combined.

The functional association between the observed QTLs on yellow rust resistance was investigated further by annotating genes detected in the QTL region using IWGSC RefSeq Annotation v1.1. Annotation revealed numerous resistance-associated genes involved in the plant defense system (**Supplementary Table 5**). Some of the identified high-potential genes implicated in the defense response to fungi include *TraesCS1B02G067000* on chromosome 1B, *TraesCS2A02G298600*, *TraesCS2A02G538200*, *TraesCS2A02G533700*, and *TraesCS2A02G538200* on 2A; *TraesCS2B02G371600* on 2B; *TraesCS2D02G470400 and TraesCS2D02G470500* on 2D; *TraesCS3A02G067600, TraesCS3A02G475500, TraesCS3A02G504700*, and *TraesCS3A02G475600* on 3A; *TraesCS5B02G245800* on 5B; *TraesCS6B02G016000, TraesCS6B02G118100*, and *TraesCS6B02G119900* on 6B; and *TraesCS7A02G286700, TraesCS7A02G441900, TraesCS7A02G442100, TraesCS7A02G442200, TraesCS7A02G442300, TraesCS7A02G448400*, and *TraesCS7B02G340500* on 7A. Furthermore, high-confidence candidate genes important for systemic acquired resistance (SAR) have been identified, revealing wheat’s long-lasting, broad-spectrum resistance to pathogen infections. These include *TraesCS2B02G57580, TraesCS2B02G581800*, and *TraesCS2B02G583600* on 2B; *TraesCS2D02G504200* on 2D; *TraesCS6A02G394900* on 6A; and *TraesCS6B02G015000* on 6B. Zooming into the significant QTL region identified high-confidence gene *TraesCS2D02G470400, TraesCS2D02G505400*, and *TraesCS2D02G470500* on 2D; *TraesCS3A02G475600* on 3A; *TraesCS6B02G015000* and *TraesCS6B02G119900* on 6B; and *TraesCS7A02G286700* on 7A, which regulate mitogen-activated protein kinase (MAPK) cascades, which are involved in signaling a variety of plant defense responses against pathogen infections (**Supplementary Table 5**).

KnetMiner (http://knetminer.rothamsted.ac.uk) searched for gene networks associated with stripe rust resistance and discovered 137 candidate genes linked to the 37 previously reported QTLs (**Supplementary Table 6**). Based on these searches, several possible genes, such as TUD1 (*TraesCS2A02G298600*), C2 (*TraesCS2A02G501300*), SARD1 (*TraesCS6B02G119900*), and CW9 (*TraesCS7B02G449900*), are associated with the regulation of systemic acquired resistance and salicylic acid-mediated signaling. Additionally, *TraesCS1B02G066300*, *TraesCS3A02G035700*, and *TraesCS3A02G500900* are involved in stripe rust reaction types. Furthermore, RGA3 (*TraesCS7B02G002300*) was recorded to a region linked with stripe rust resistance, reaction type T1, seedling resistance, severity T1, systemic acquired resistance, and salicylic acid-mediated signaling (**Supplementary Table 6**).

## 4 Discussion

### 4.1 Population structure, relatedness, and LD

The existence of three subpopulations (K=3) with significant admixture was supported by population structure and principal component analyses. Gao et al. (2016) presented parallel indistinct population grouping, increased admixing, and reduced population sub-structuring for bread wheat genotypes using 7k SNP markers. In addition, kinship analysis confirmed the presence of ambiguous familial relatedness, emphasizing the need to include both population structure (Q) and kinship (K) as factors in marker-trait association analyses. The BLINK model used in the association studies adequately adjusted for population stratification, relatedness, and marker effects, decreasing confounding effects that could lead to false-positive MTAs. Likewise, visualizing the Q–Q plots confirmed the effective control of the confounding elements. Overall, the combination of population structure and kinship analysis, as well as the usage of BLINK model, has increased the accuracy and reliability of marker-trait associations.

In terms of marker distribution across chromosomes, the bread wheat sub-genomes were fairly distributed, with the A sub-genome contributing the most, B sub-genome coming in second and D sub-genome harboring the fewest. This suggests that the A sub-genome may have undergone more extensive evolutionary changes than the B and D sub-genomes. Comparable findings were published by Kokhmetova et al. (2023), who found that the A sub-genome contributes the most, the B sub-genome comes in second, and the D sub-genome ports the least. Furthermore, this could indicate that the A and B sub-genomes had a disproportionately high number of SNPs, which was most likely caused by the D-genome’s more recent incorporation into the hexaploidy wheat genome. In addition, the LD varied among the three sub-genomes, with the A and B sub-genomes having lower LD, possibly due to their longer evolutionary histories than the D genome. Likewise, discrepancies in LD between sub-genomes could be similar to genetic drift and selection factors that have influenced their evolutionary paths through time. This fluctuation in LD demonstrates the complicated genetic mechanisms at work inside the wheat genome.

### 4.2 Marker-trait associations and identification of candidate genes

The identification of substantial marker-trait associations of disease severity, seedling response, and coefficient of infection at a significance level of nominal p-value = 0.001 revealed 102 marker-trait associations pointing to 44 quantitative trait loci. Previously, GWAS analysis for yellow rust resistance identified marker-trait associations (MTAs) using various genetic panels and marker systems (Long et al., 2019; Franco et al., 2022; Khan et al., 2024). For instance, Khan et al. (2024) found 24 MTAs in bread wheat genotypes. The large sample size and SNPs used in the current study could be the primary factors of the significant variation in the number of MTAs. Moreover, it could be due to the extensive genetic diversity present in the bread wheat genotypes studied, contributing to the identification of numerous marker-trait associations.

Among the 102 discovered MTAs, 31 (30.39%) conferred yellow rust disease severity resistance, 41 (40.20%) conferred seedling resistance, and 30 (29.41%) conferred infection coefficient resistance. These results suggest that different MTAs may play distinct roles in providing resistance to yellow rust disease in different traits. On the other hand, genome-wide scans for yellow rust resistance for each isolate revealed several indicators related to yellow rust resistance across isolates. MTA analysis revealed 19 MTAs in the Dekaa isolate, 25 in the Hdasee isolate, 27 in the Meraro isolate, and 22 in the Sanate isolate. This suggests that various isolates may have distinct genetic variables leading to yellow rust resistance, emphasizing the need to include isolate-specific markers in breeding programs. Parallelly, Atsbeha et al. (2023) investigated adult plant resistance and discovered 48 yellow rust resistance SNPs to be environment-specific (at Meraro), indicating the presence of *P. striiformis* races at the test site that differed from other test sites, as well as race-specific resistance genes. This demonstrates that the genetic response to yellow rust varies according to the pathogen’s race.

Comparison of genome wide LD decay in the panel with previous studies in wheat revealed that it (~4.2 Mb) is in the range (4–8 Mb) reported for highly diverse wheat germplasm sets (Liu et al., 2017; Ladejobi et al., 2019; Li et al., 2019; Krishanpa et al., 2022; Rathan et al., 2022; Sehgal et al., 2024). The average LD decay is faster than obtained in germplasm from Kazakhstan (22Mb; Kokhmetova et al., 2021) or Mexican bread wheat landraces (Vikram et al., 2021). The high diversity of the panel is due to inclusion of CIMMYT breeding lines, consisting of lines selected from a wide range of genetic backgrounds (Sehgal et al., 2020). Following are the cited papers that I added here. Please update them in the reference list.

LD criteria were used to allocate the 102 MTA markers to 44 putative QTLs. As a result, the association analysis for yellow rust resistance in individual isolates identified 25 potential QTLs covering all three sub-genomes. It was discovered that some of the revealed putative QTLs were beneficial against yellow rust in more than two isolates. This implies that these QTLs may have a broad-spectrum effect on yellow rust resistance, making them potentially valuable targets for breeding efforts aimed at enhancing resistance in wheat varieties. Although it is difficult to compare the positions of QTLs from diverse studies due to variation in mapping methodologies, marker systems, and mapping populations used, some QTLs discovered in this study coincided with the mapping positions of previously reported yellow rust-resistant genes in the literature. Several previous studies reported yellow rust-resistant QTLs on 1B (Jamil et al., 2020; Alemu et al., 2020; Baranwal et al., 2022; Kokhmetova et al., 2023), 2A (Gebrewahid et al., 2020; Zhang et al., 2021; Baranwal et al., 2022), 2B (Li et al., 2020; Kumar et al., 2020; Rauf et al., 2022), 2D (Kumar et al., 2022; Baranwal et al., 2022), 3A (Kokhmetova et al., 2023; Atsbeha et al., 2023), 3B (Kumar et al., 2020; Rauf et al., 2022; Kumar et al., 2022; Atsbeha et al., 2023), and 7A (Kokhmetova et al., 2023). However, seven of the 44 detected QTLs (qYrSe.10 on chromosome 2B, qYrSe.14 and qYrSe.15 on chromosome 2D, qYrSe.19 on chromosome 3A, qYrSe.25 on chromosome 3D, and qYrSe.36 and qYrSe.37 on chromosome 7A) were not reported in previous wheat literature and the International Wheat Genome Sequencing Consortium could be potentially novel QTLs.

The seven potentially new have QTL frequency ranges of 56%–88% for the SR_Sanate and DS_Hdasee, respectively (**Supplementary Table 7**). This high frequency of the QTLs highlights their potential importance in conferring resistance to stripe rust in the studied bread wheat populations. The frequency range suggests that these QTLs were widely present in the tested population, indicating their potential effectiveness in combating the Dekaa, Hdasee, Meraro, and Sanate isolate. This QTL importance in disease severity, seedling response, and coefficient of infection traits indicates that they could be useful breeding targets for developing durable, stripe rust-resistant wheat varieties.

Additionally, the impact of potentially new QTLS varies from 0.57 to −5.32. DS_Dekaa and SR_Sanate had the highest positive and negative impacts, respectively, whereas SR_Meraro and CI_Meraro had the least positive and negative effects. The positive and negative effects indicate that certain QTLs may enhance the trait whereas others suppress it, emphasizing the complexities of genetic factors influencing stripe rust resistance. Furthermore, many genotypes such as G1, G3, G8, G10, G14, G92, G98, G102, and G178 contain all potentially novel QTLs for all traits (**Supplementary Table 7**). This implies that these genotypes with all potentially novel QTLs for all traits could be valuable resources for breeding programs aiming to develop new varieties with improved stripe rust resistance.

A recent GWAS conducted by Franco et al. (2022) found substantial variation in resistance loci, which contributed to the diversification of resistance genes and advancements in durable disease resistance strategies. Similarly, our study identified multiple QTLs associated with yellow rust resistance across various isolates, reinforcing the role of genetic diversity in conferring resistance to stripe rust. Specifically, some of the stable QTLs identified in our study (e.g., qYrSe.04, qYrSe.07, and qYrSe.12) were effective for resistance across different isolates, much like the loci identified in the Argentine study, which contributed to durable resistance in local wheat cultivars. Another GWAS conducted on 245 spring bread wheat genotypes in Argentina identified several QTLs associated with resistance to local *Puccinia striiformis* f. sp. tritici races, further demonstrating the genetic underpinnings of stripe rust resistance. The study revealed specific QTLs that were strongly associated with resistance to these local races, contributing to the broader understanding of wheat’s defense mechanisms (Franco et al., 2022).

In another study, Maccaferri et al. (2015) conducted a GWAS on hexaploid spring wheat from around the world, identifying resistance loci on chromosomes 1B, 3B, and 5A. These findings are consistent with our study, where we detected QTLs on chromosomes 3B and 5A, indicating the conservation of resistance loci across different wheat populations. These studies underscore the importance of global collaboration in understanding the genetic mechanisms underlying stripe rust resistance. The loci identified in our research, especially those on chromosomes 3B and 5A, may serve as targets for marker-assisted selection and pyramiding efforts in wheat breeding. Furthermore, a study by Gur et al. (2022) identified multiple MTAs for stripe rust resistance in wild emmer wheat (*Triticum turgidum* subsp. *dicoccoides*). This study reported several MTAs on chromosomes 2A, 3A, and 7B, which were also found in our study to be associated with yellow rust resistance. The consistent identification of resistance loci on chromosomes 2A, 3A, and 7B across different wheat populations and species highlights the potential role of these genomic regions in providing broad-spectrum resistance to stripe rust.

*TraesCS1B02G067000* on chromosome 1B, *TraesCS2A02G538200* on 2A, *TraesCS2B02G371600* on 2B, *TraesCS2D02G470500* on 2D, *TraesCS3A02G475600* on 3A, *TraesCS5B02G245800* on 5B, *TraesCS6B02G119900* on 6B, and *TraesCS7B02G340500* on 7A were discovered to be significantly upregulated in response to fungal infection. Aside from systemic acquired resistance (SAR), high-confidence candidate genes have shown that wheat has long-term, broad-spectrum resistance to pathogen infections. These are *TraesCS2B02G583600* on 2B, *TraesCS2D02G504200* on 2D, *TraesCS6A02G394900* on 6A, and *TraesCS6B02G015000* on 6B. This implies that wheat has a sophisticated genetic network that allows it to protect against a variety of infections, including fungal disease. As an illustration, RGA3 (*TraesCS7B02G002300*) reported in on chromosome 7B, qYrSe.40 plays a crucial role in plant defense mechanisms, particularly in response to stripe rust infection. This gene has been identified as being involved in multiple aspects of stripe rust resistance, including reaction type T1, seedling resistance, and severity T1. One of the key defense strategies associated with RGA3 is systemic acquired resistance (SAR), a broad-spectrum immune response that enables plants to develop long-lasting resistance against various pathogens (Klessig et al., 2018). SAR is typically mediated by the salicylic acid (SA) signaling pathway, which plays a critical role in enhancing the plant’s ability to recognize and counteract infections. The repeated mention of systemic acquired resistance (SAR) in association with RGA3 indicates its strong involvement in activating defense-related genes and promoting resistance mechanisms at both the seedling stage and later developmental phases.

Furthermore, gene *TraesCS1B02G067000*, located on chromosome 1B of bread wheat (*Triticum aestivum* L.), encodes a protein characterized by a knottin/scorpion toxin-like domain. Proteins with knottin domains, often referred to as plant defensins, are small, cysteine-rich peptides integral to the plant’s innate immune system. These defensins exhibit antimicrobial properties against a variety of pathogens, including fungi, bacteria, and viruses, and are distributed across different plant tissues (Li et al., 2021). Additionally, *TraesCS5B02G245800* located on chromosome 5B of wheat has been implicated in resistance mechanisms against stripe rust. This gene is part of a broader network of pathways that are activated in response to pathogen attack, contributing to wheat’s defense system, whereas its involvement in host–pathogen interactions and potential role expression and plant defense mechanisms. Activation of the *TraesCS5B02G245800* gene led to the upregulation of various plant defense-related genes. In response to stripe rust infection, the plant likely increases the expression of NBS-LRR (nucleotide-binding site leucine-rich repeat) resistance genes, which are key to recognizing pathogen effectors and initiating the hypersensitive response (Liu et al., 2021). This defense mechanism leads to localized cell death to limit pathogen spread and activation of broader immune responses throughout the plant.

*TraesCS2D02G470500* on 2D, *TraesCS3A02G475600* on 3A, *TraesCS6B02G119900* on 6B, and *TraesCS7A02G286700* on 7A control mitogen-activated protein kinase (MAPK) cascades play a role in signaling a range of plant defense responses to pathogen infection. These genes are essential for activating wheat defensive systems (Lefevere et al., 2020). Furthermore, gene *TraesCS2D02G470500*, located on chromosome 2D of bread wheat (*Triticum aestivum* L.), encodes a leucine-rich repeat receptor-like protein kinase (LRR-RLK). LRR-RLKs are pivotal in plant defense, primarily by recognizing pathogen-associated molecular patterns (PAMPs) and activating downstream defense responses. Upon recognition of PAMPs, LRR-RLKs initiate signaling cascades, notably the mitogen-activated protein kinase (MAPK) pathways. These MAPK cascades transmit extracellular signals to intracellular responses, leading to the activation of defense genes and the production of antimicrobial compounds. In the context of stripe rust, MAPK pathways play a significant role in mediating resistance. For instance, the wheat gene TaMAPK4 has been identified as a positive regulator in defense against stripe rust. Silencing of TaMAPK4 results in increased susceptibility to Pst, highlighting its importance in the defense mechanism (Krasileva et al., 2016; Wang et al., 2018).

## 5 Conclusion

Stripe rust caused by the fungus *P. striiformis* in Ethiopia is one of the most economically important diseases of wheat, leading to significant yield losses each year. Wherein, farmers face significant yield losses and economic hardship due to the disease’s impact on wheat crops. Use of resistant varieties is an effective and environmentally friendly strategy for managing stripe rust in wheat fields. Genome-wide association analysis is an influential means used to identify genetic variations associated with complex traits like disease resistance by associating the genomes of plants with the disease. In this study, genomic regions underlying yellow rust resistance in seedling plants were investigated using genome-wide scanned SNPs and isolates derived from phenotype data in a bread wheat association panel. The study also found 102 SNPs that were significantly associated with strong yellow rust resistance, pointing to 44 QTLs. Since some of these putative QTLs were stable across all isolates, they may be regarded as the main genomic loci that contain gene combinations that give all isolates resistance to *P. striiformis.* Furthermore, functional dissection of the detected QTL region in the wheat database revealed several defense-related candidate genes involved in plant resistance to fungal infections, systemic acquired resistance, and MAPK pathways that signal a variety of plant defense systems. The majority of the discovered putative QTLs had similar chromosomal locations to previously described genes; however, seven QTLs were potentially unique. These seven QTLs can be exploited in wheat resistance breeding efforts to develop broad-spectrum and long-lasting resistant wheat varieties to stripe rust. Moreover, these seven newly identified QTLs could be used for marker-assisted selection (MAS), genomic selection (GS), and gene editing in breeding programs to develop wheat varieties with enhanced resistance to fungal pathogens.

## Data Availability

The datasets presented in this study can be found in online repositories. The names of the repository/repositories and accession number(s) can be found in the article/**Supplementary Material**.
