# Supplementary material for: Genome-wide association study of seedling–plant resistance to stripe rust in bread wheat (Triticum aestivum L.) genotypes
Source: Front Plant Sci. 2025 May 2;16:1554216. doi: 10.3389/fpls.2025.1554216 (PMC12081426; doi:10.3389/fpls.2025.1554216)
Supplement: Supplementary file 1 [file Table1.doc]

**
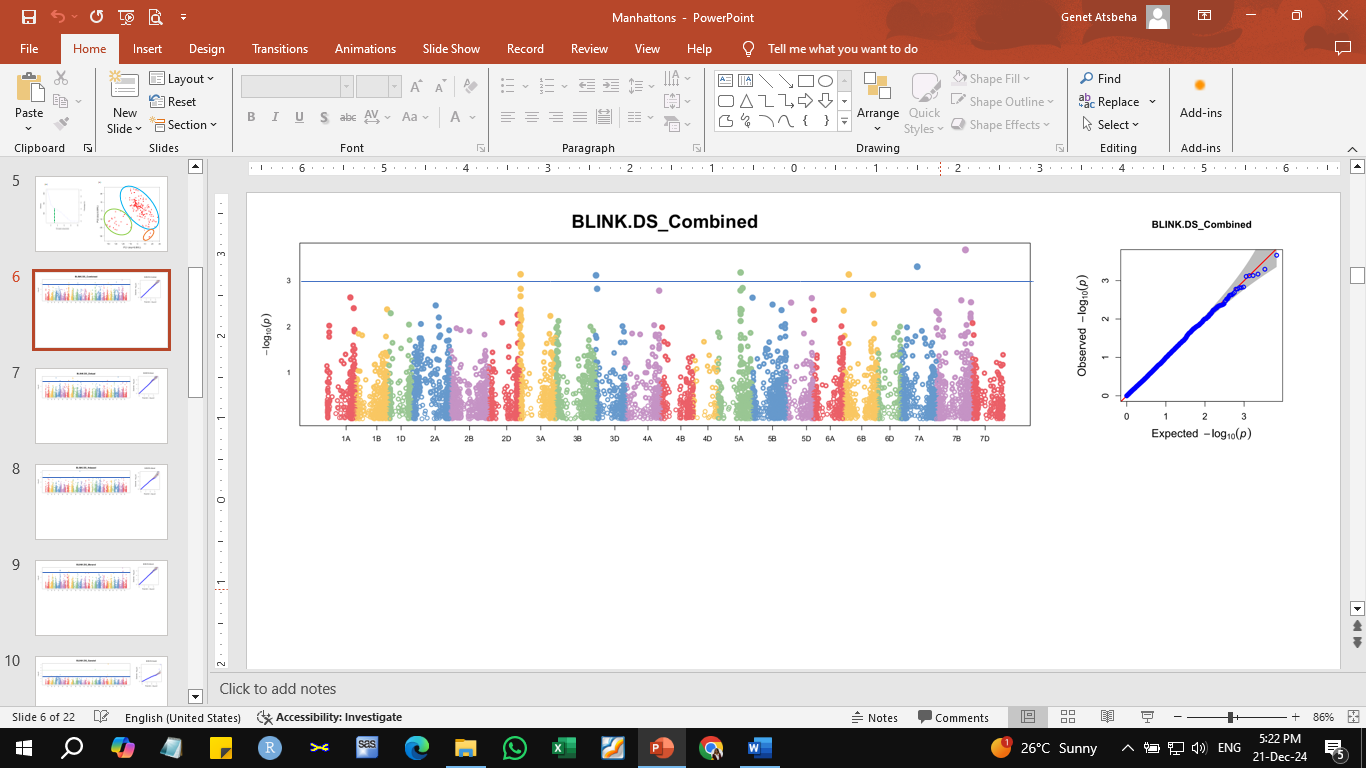
**

**
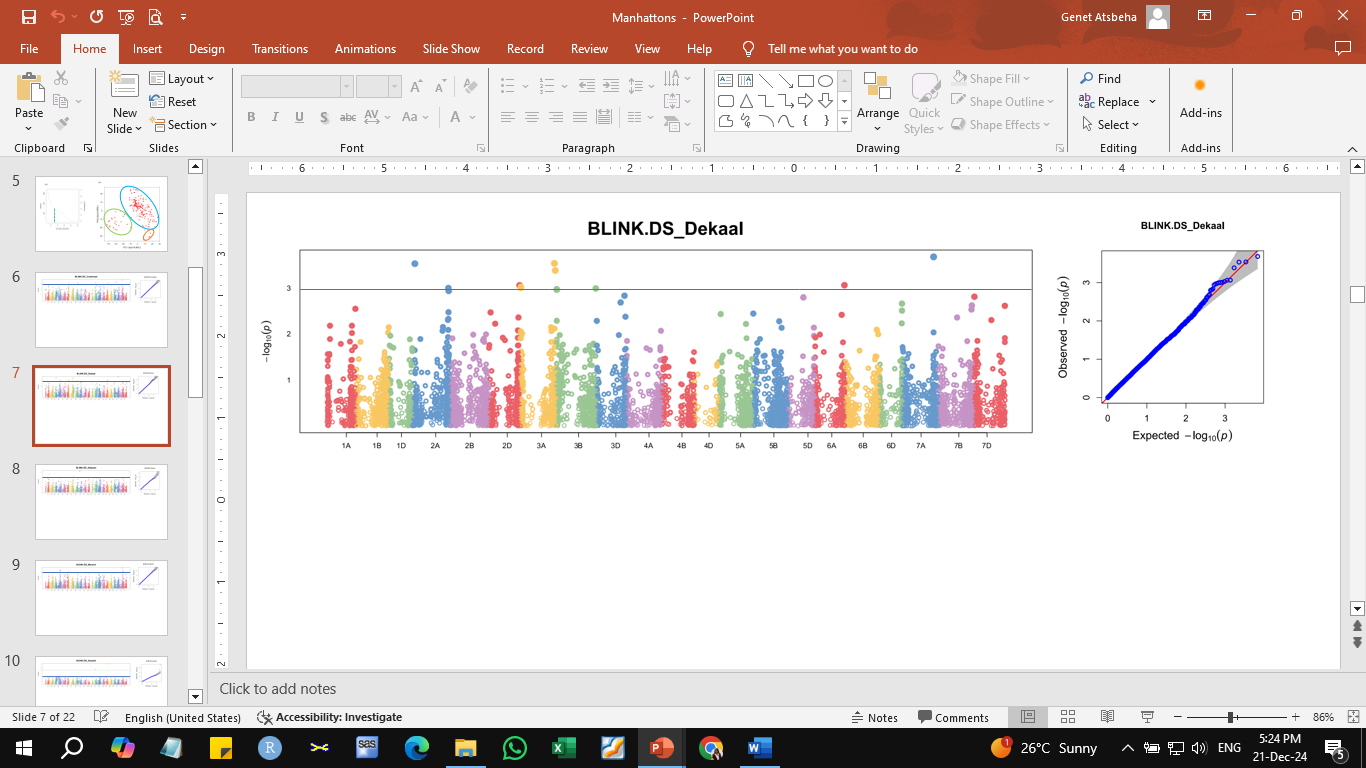
**

**
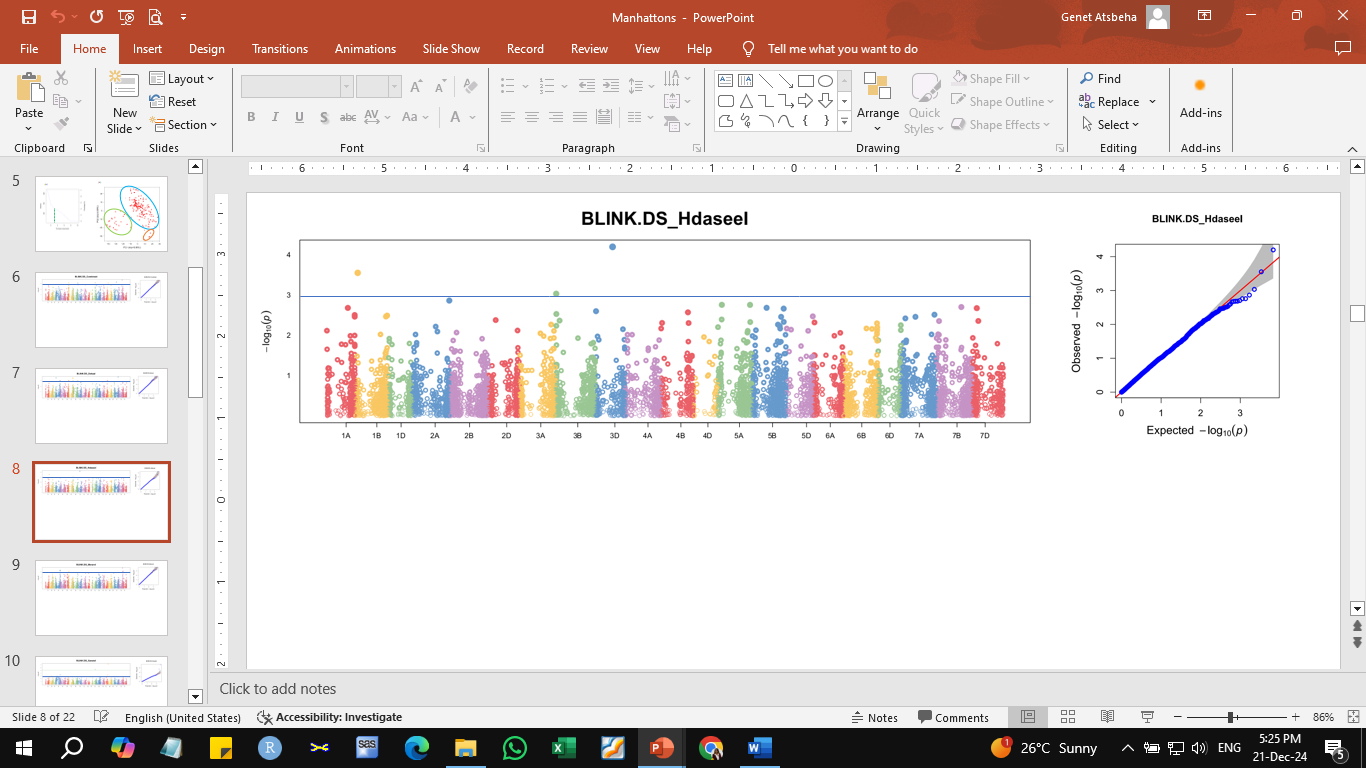
**

**
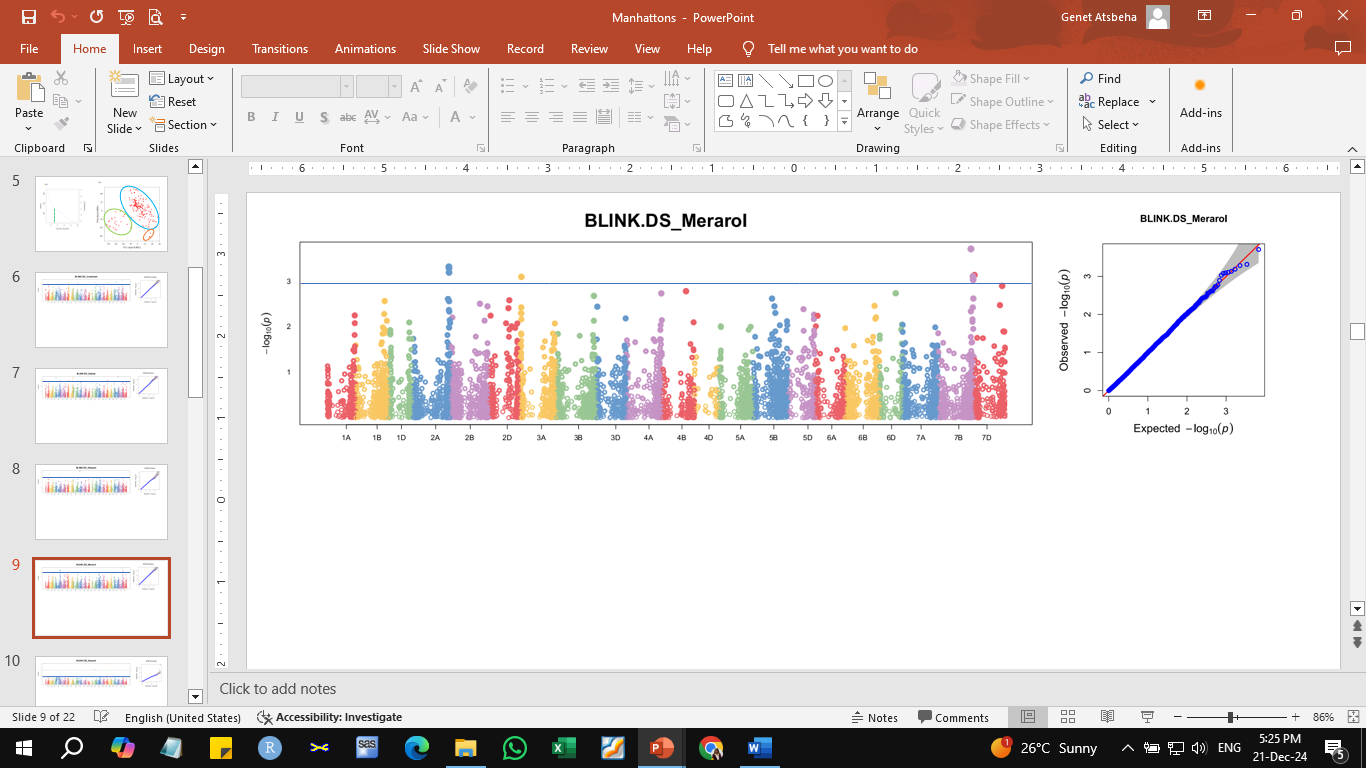
**

**
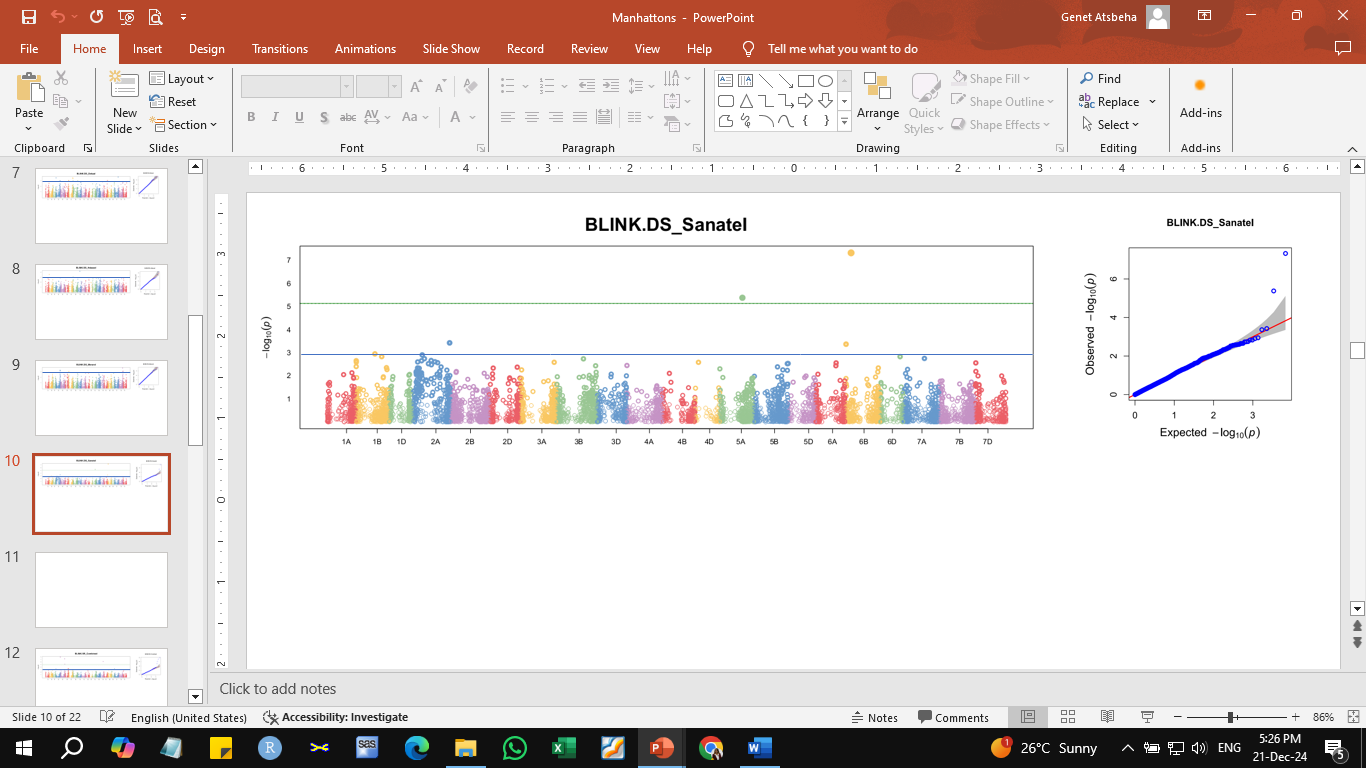
**

**
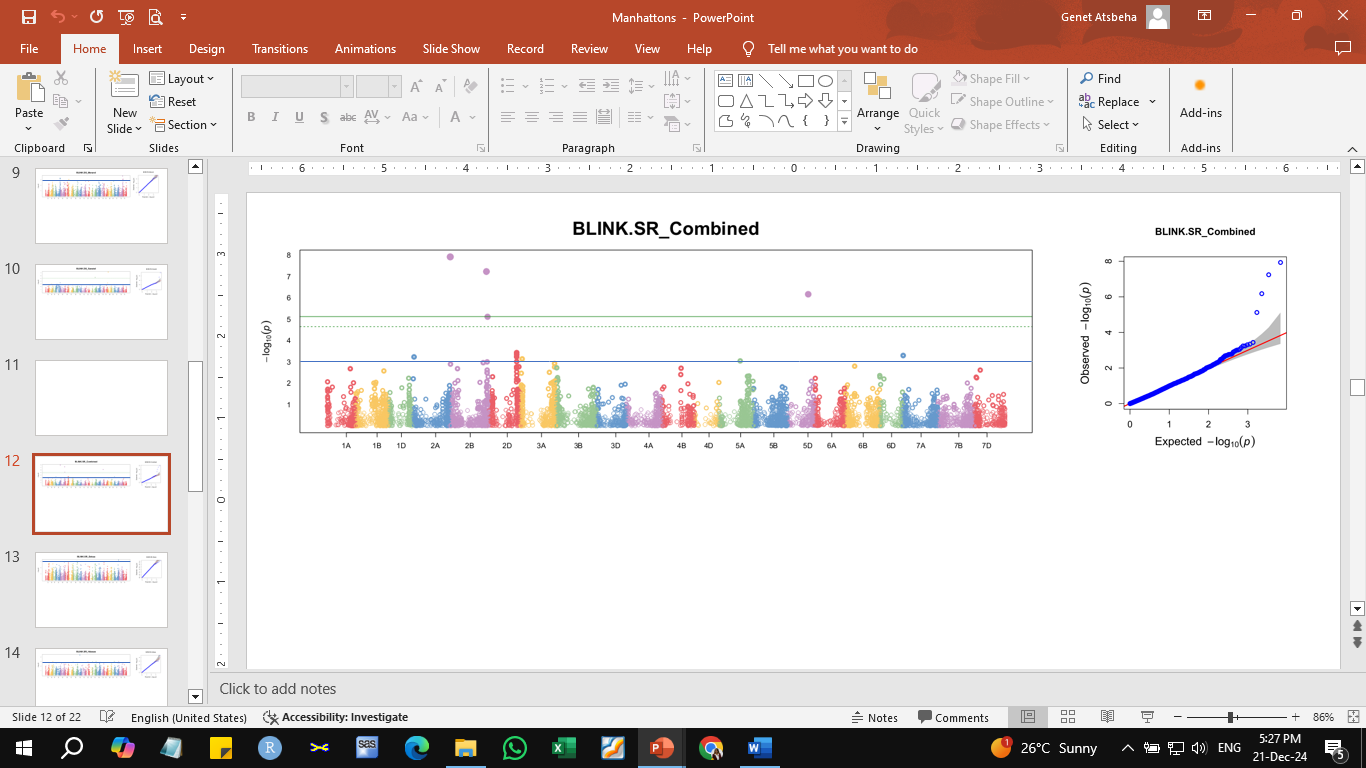
**

**
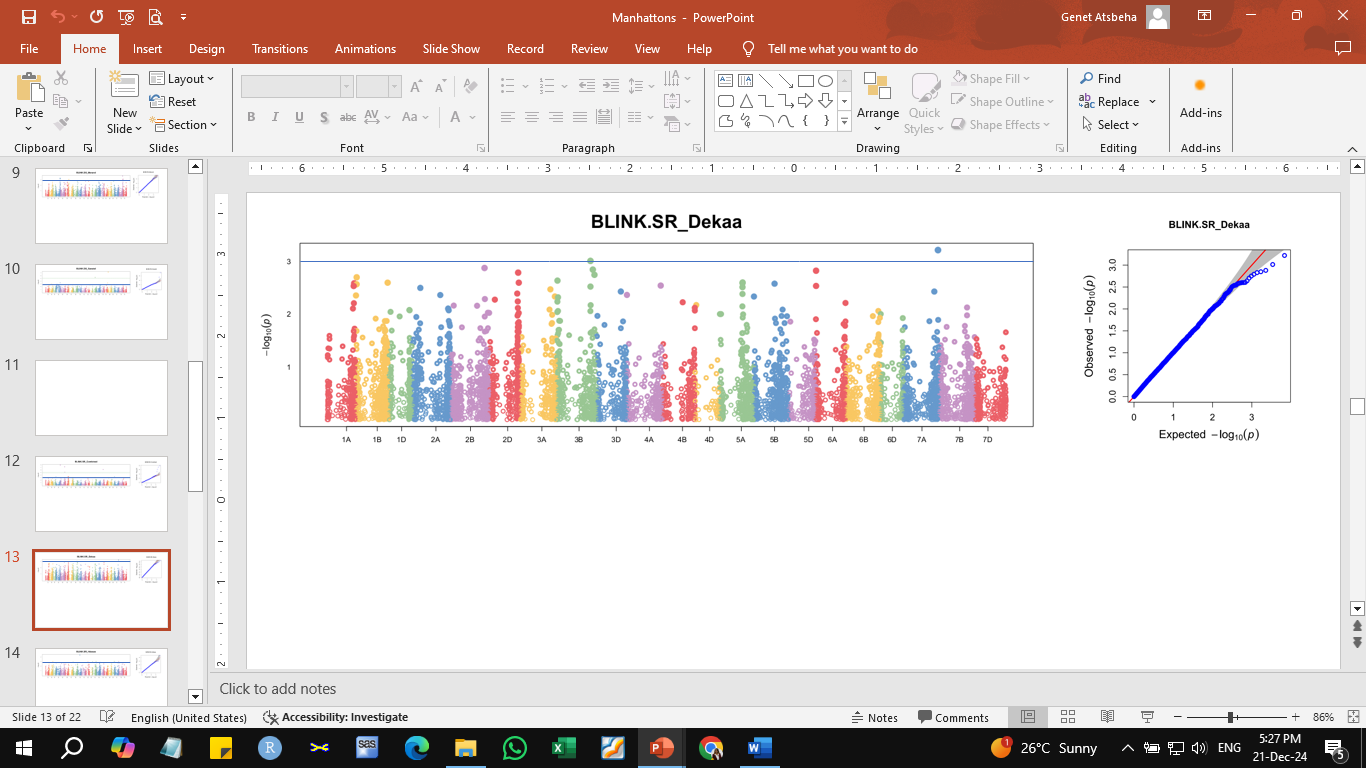
**

**
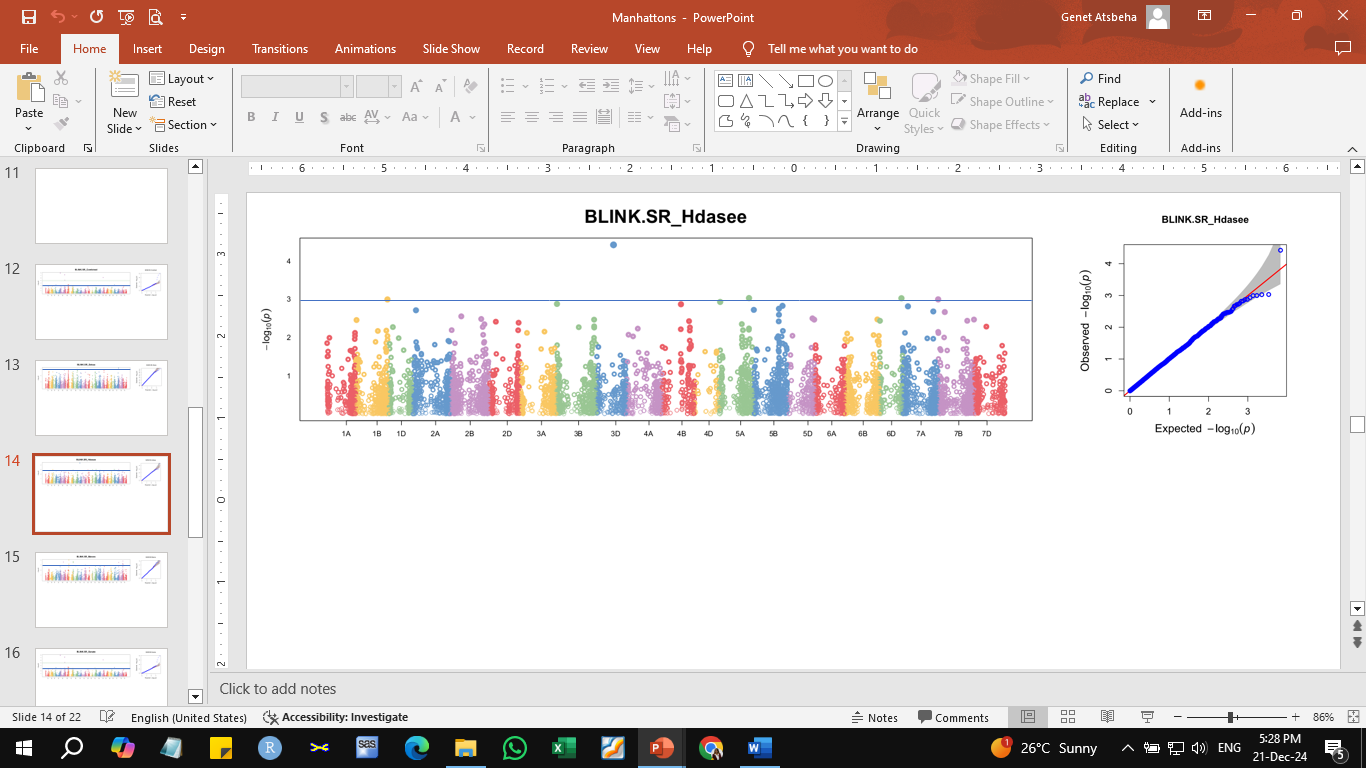
**

**
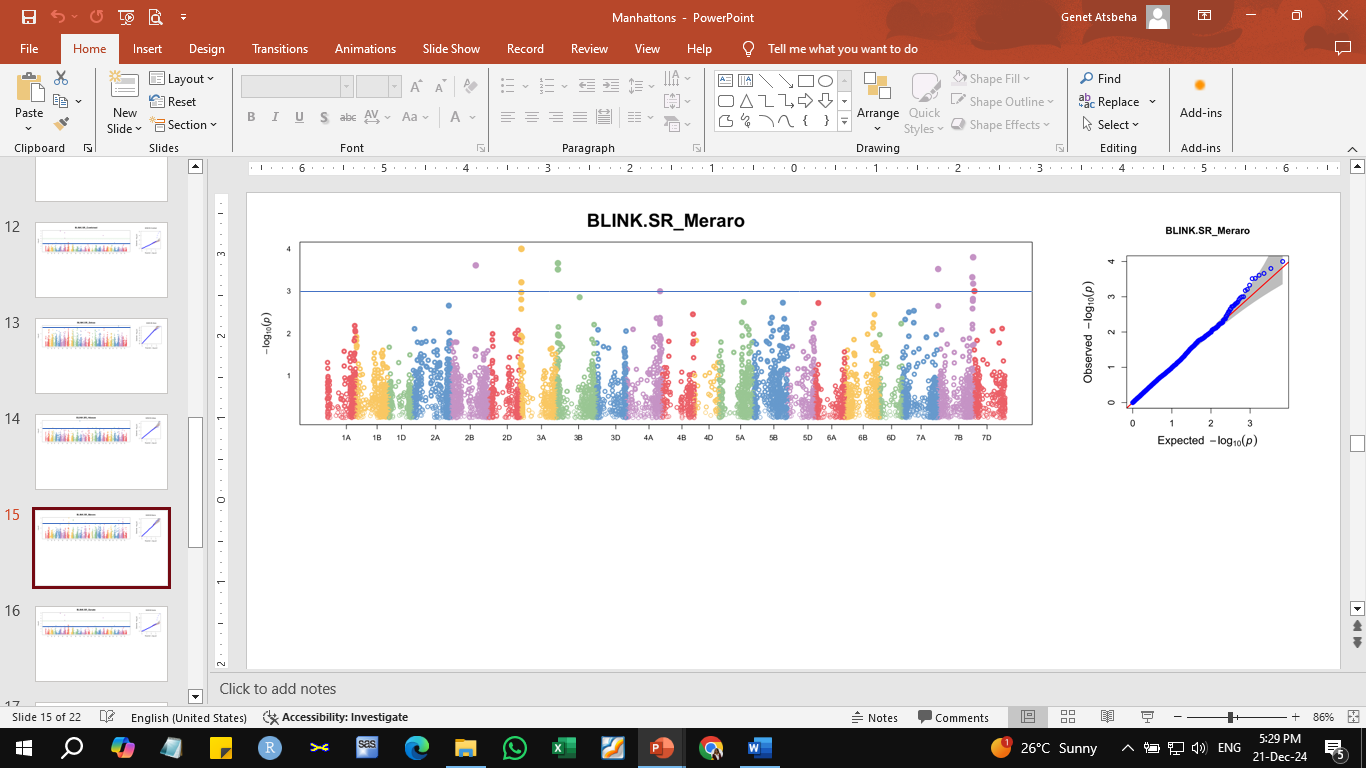
**

**
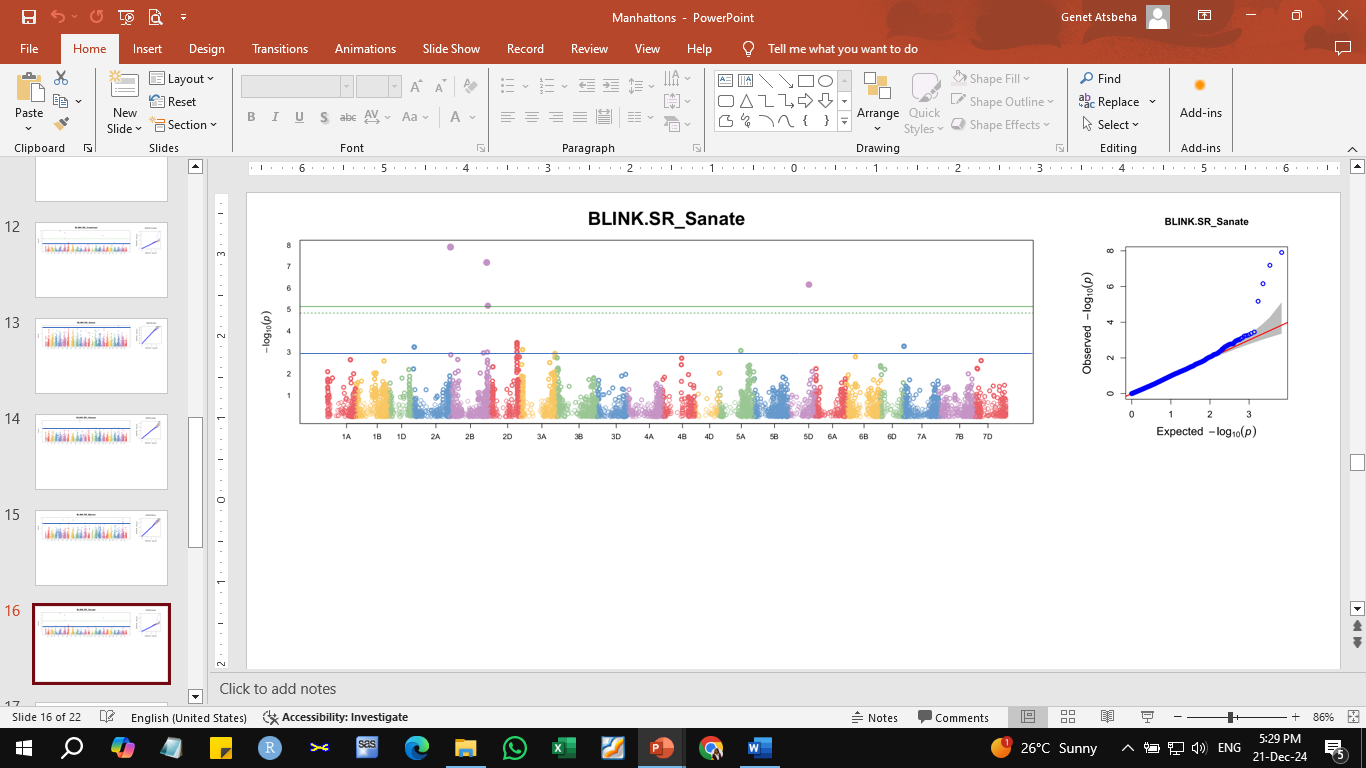
**

**
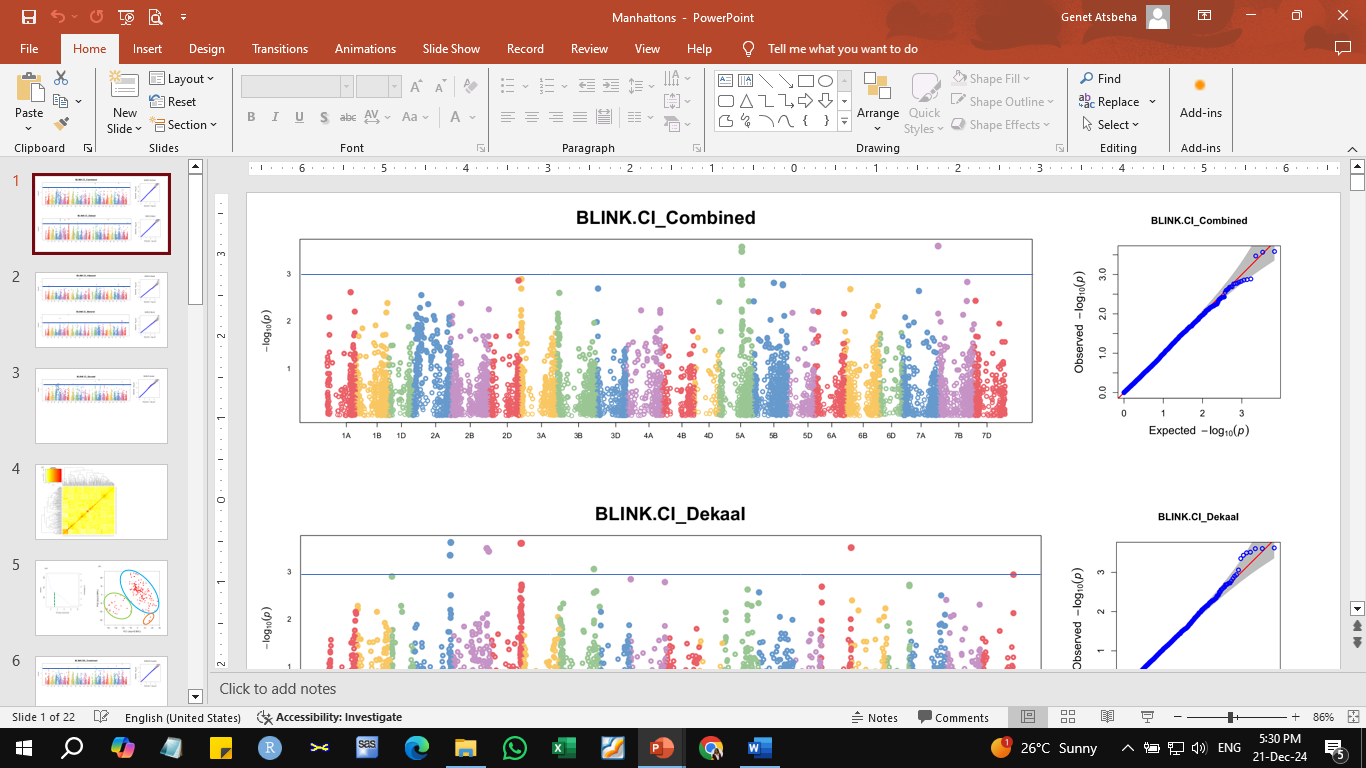
**

**
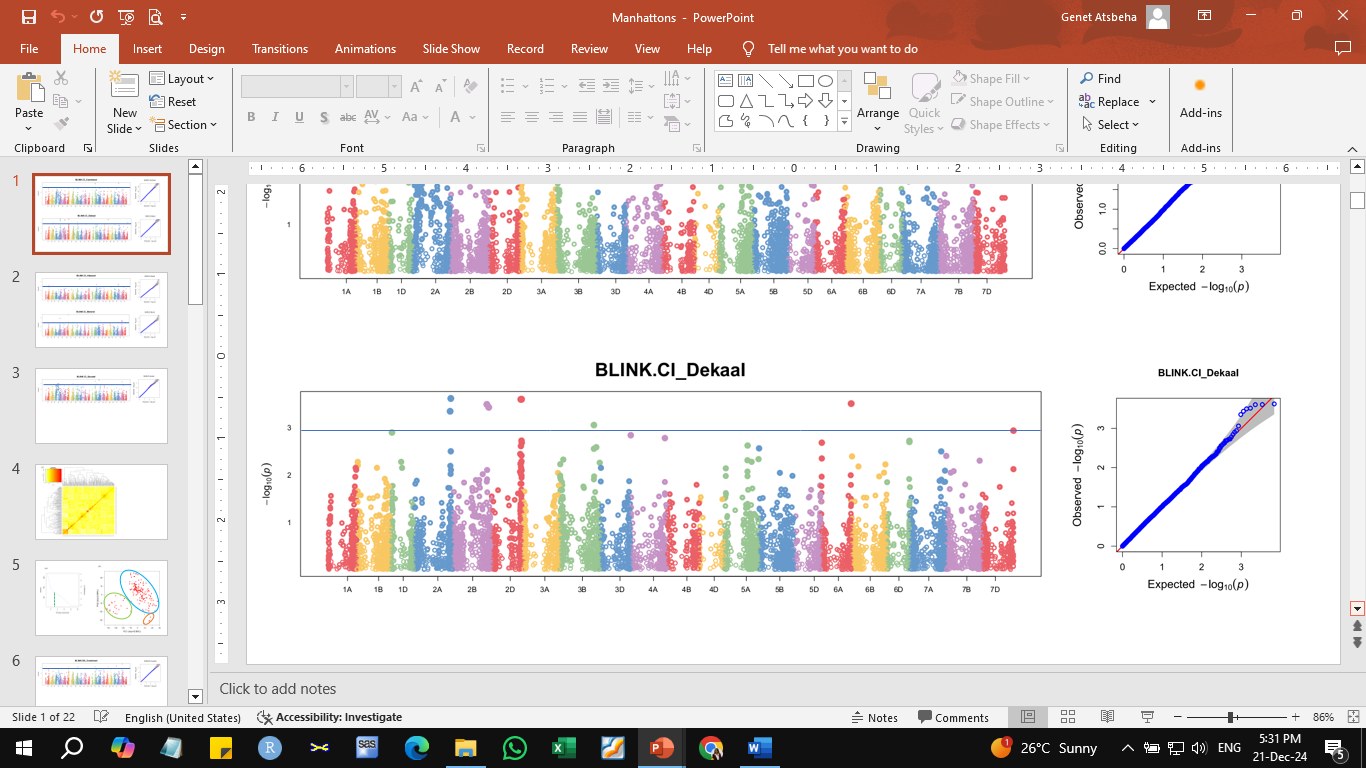
**

**
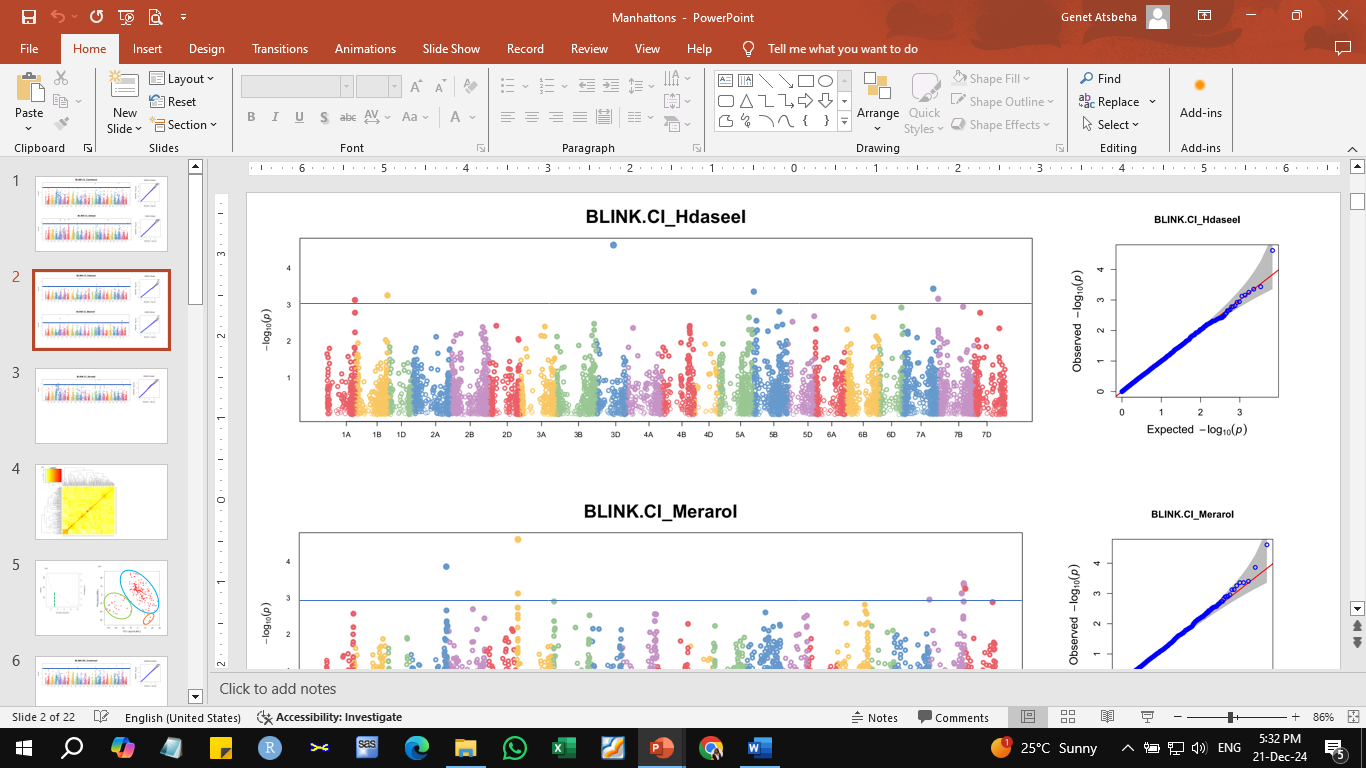
**

**
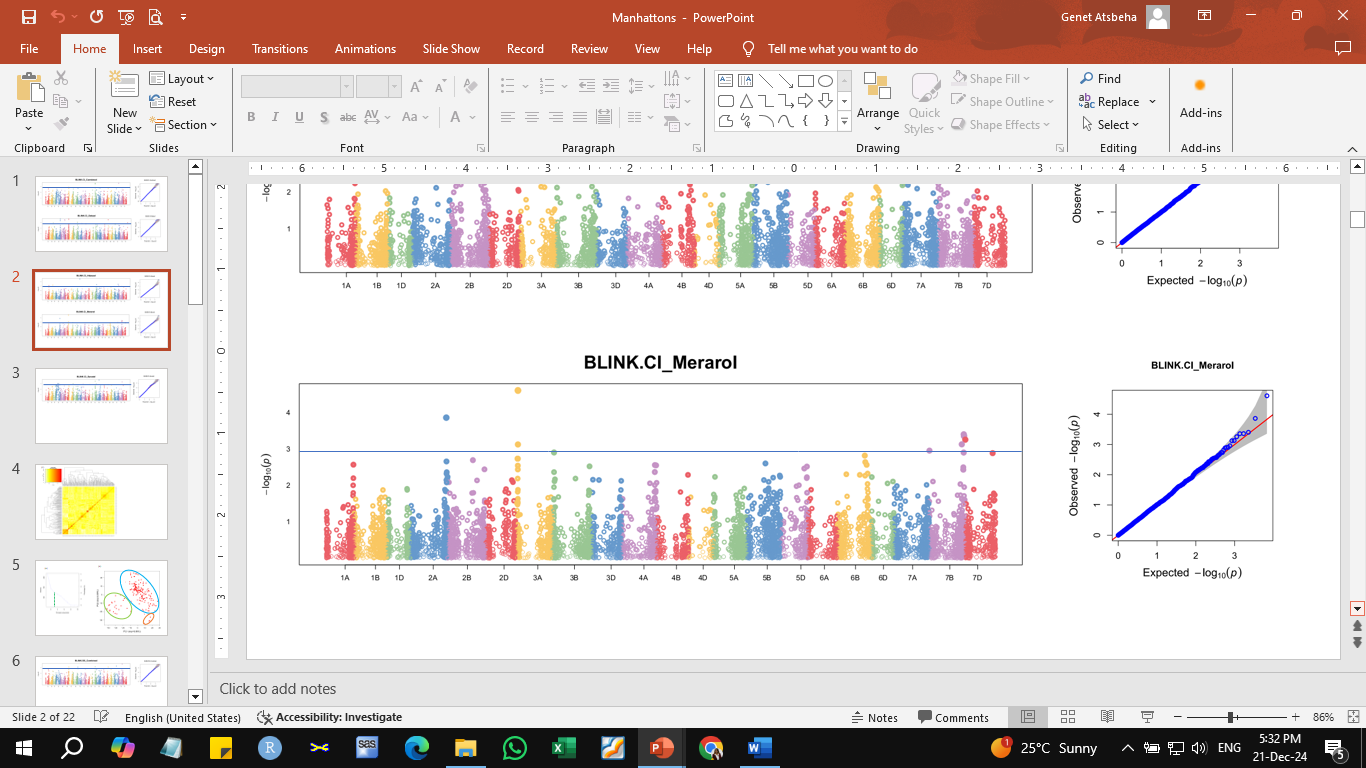
**

**
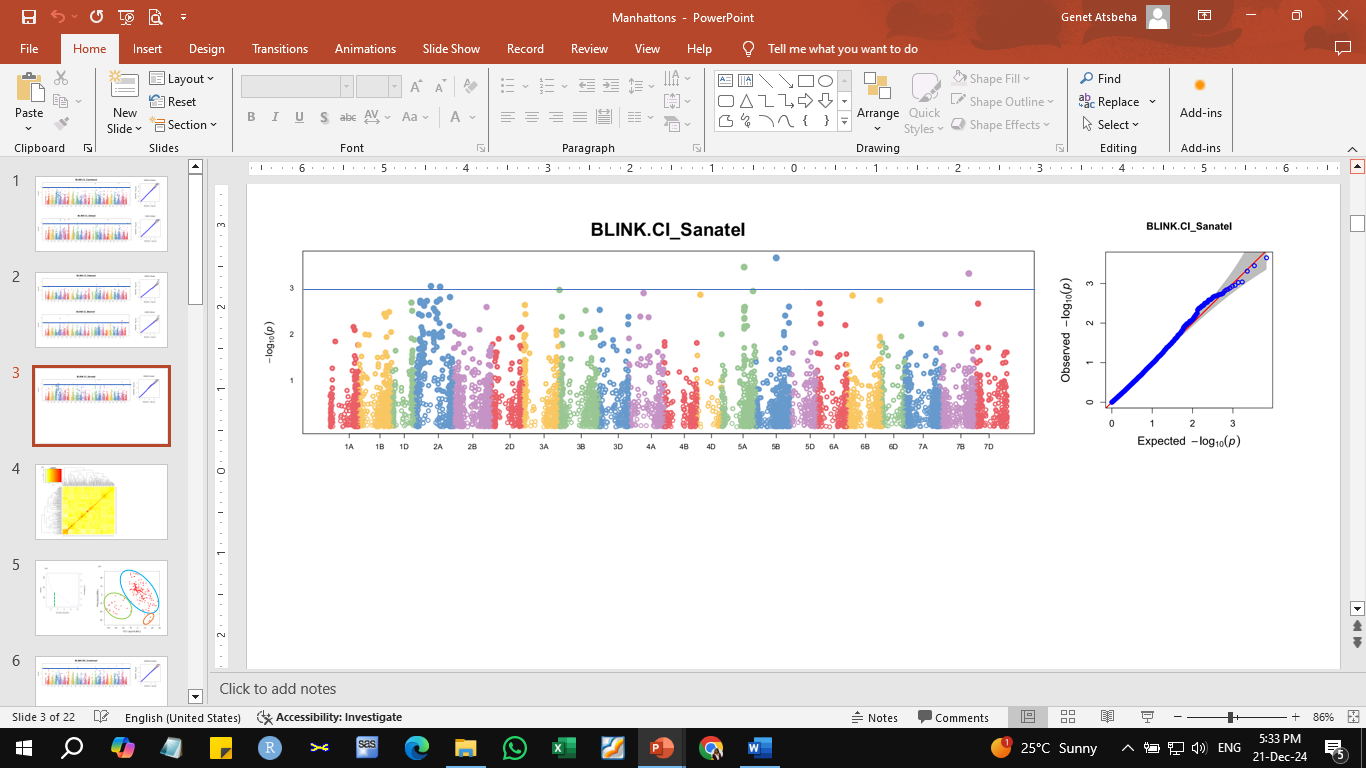
**

**Supplementary figure 1**. Manhattan plots for yellow rust traits. GWAS scans resulting with significant associations. Each dot represents a SNP. On the x-axis is the genomic position of the SNPs on the corresponding chromosomes indicated in different colors. On the “y”axis is the *-log10* of the *P-value* depicting the significance of the association test. The horizontal orange line is the nominal *p* value 0.001 significance threshold used in the association studies of DS_DekaaI = Disease severity for Dekaa isolate, DS_HdaseeI = Disease severity for Hdasee isolate, DS_MeraroI = Disease severity for Meraro isolate, DS_SanateI = Disease severity for Sanate isolate, DS_Combined = Disease severity Combined. SR_DekaaI = Seedling response for Dekaa isolate, SR_HdaseeI = Seedling response for Hdasee isolate, SR_MeraroI = Seedling response for Meraro isolate, SR_SanateI = Seedling response for Sanate isolate, SR_Combined = Seedling response Combined, CI_DekaaI = Confident of infection for Dekaa isolate, CI_HdaseeI = Confident of infection for Hdasee isolate, CI_MeraroI = Confident of infection for Meraro isolate, CI_SanateI = Confident of infection for Sanate isolate, CI_Combined = Confident of infection Combined. The quantile-quantile (Q-Q) plots at the right side of the Manhattan plots indicate how well the used BLINK model accounted for population structure and kinship for each of the disease traits. In each plot, the observed *–log (P values)* from the fitted GWAS models (y-axis) are compared with their expected value (x-axis) under the null hypothesis of no association with the trait. Each blue dot represents a single nucleotide polymorphism; the red line is the model for no association.
