# Supplementary material for: Genome-wide association study of seedling–plant resistance to stripe rust in bread wheat (Triticum aestivum L.) genotypes
Source: Front Plant Sci. 2025 May 2;16:1554216. doi: 10.3389/fpls.2025.1554216 (PMC12081426; doi:10.3389/fpls.2025.1554216)
Supplement: Supplementary file 2 [file Table2.doc]

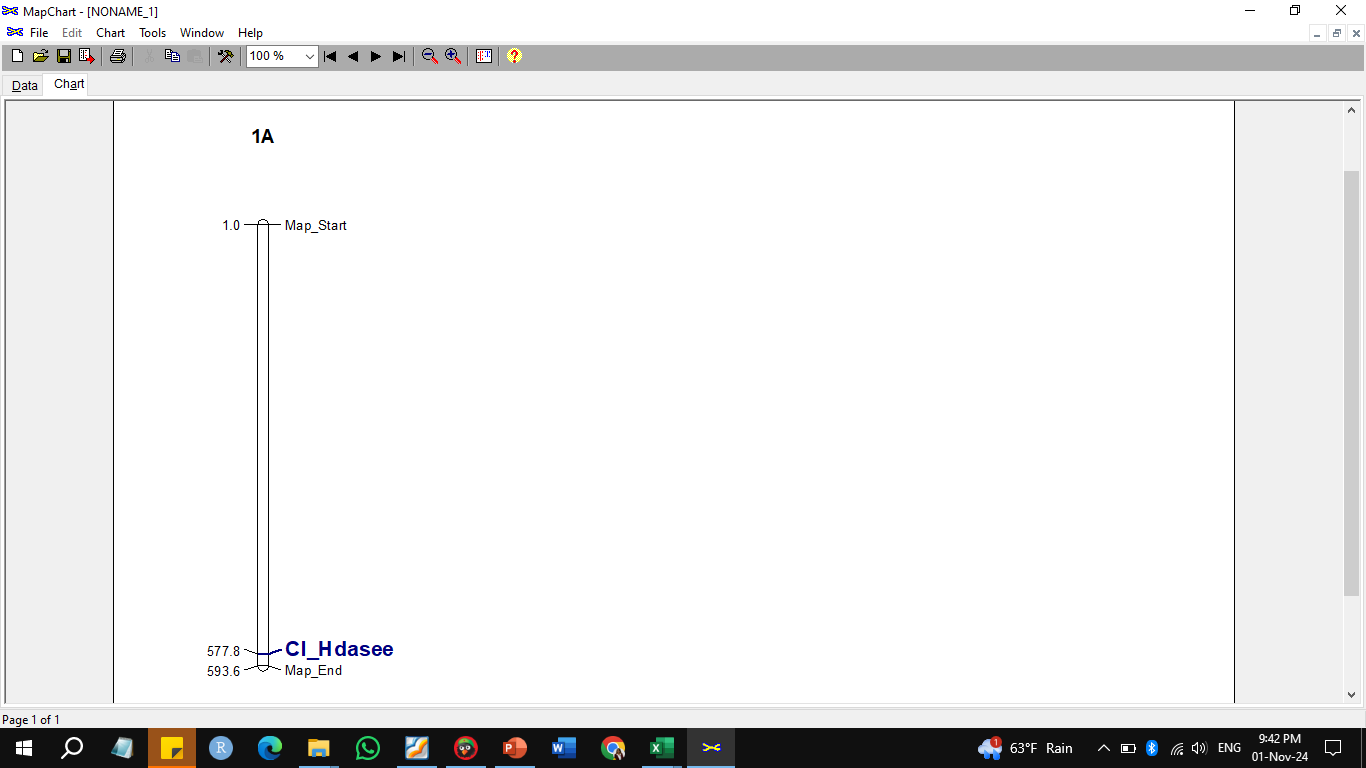

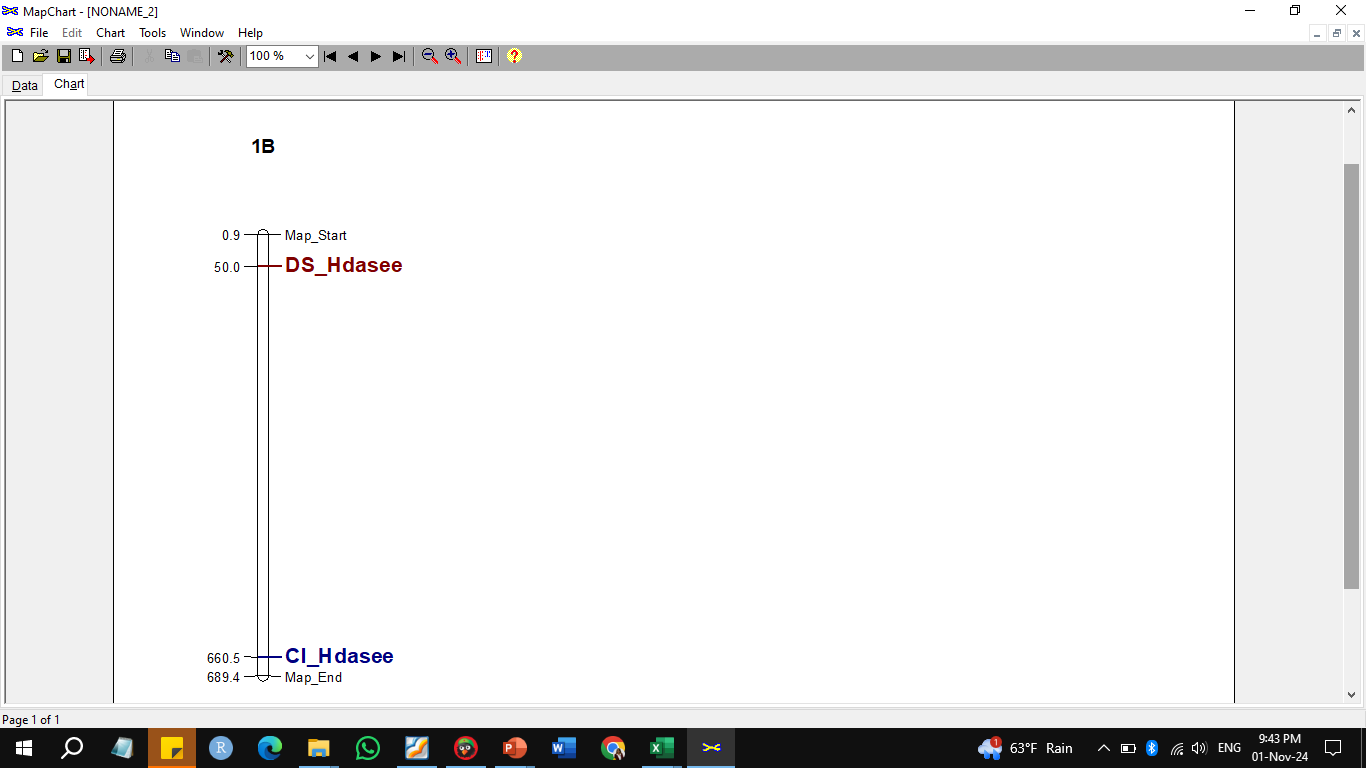

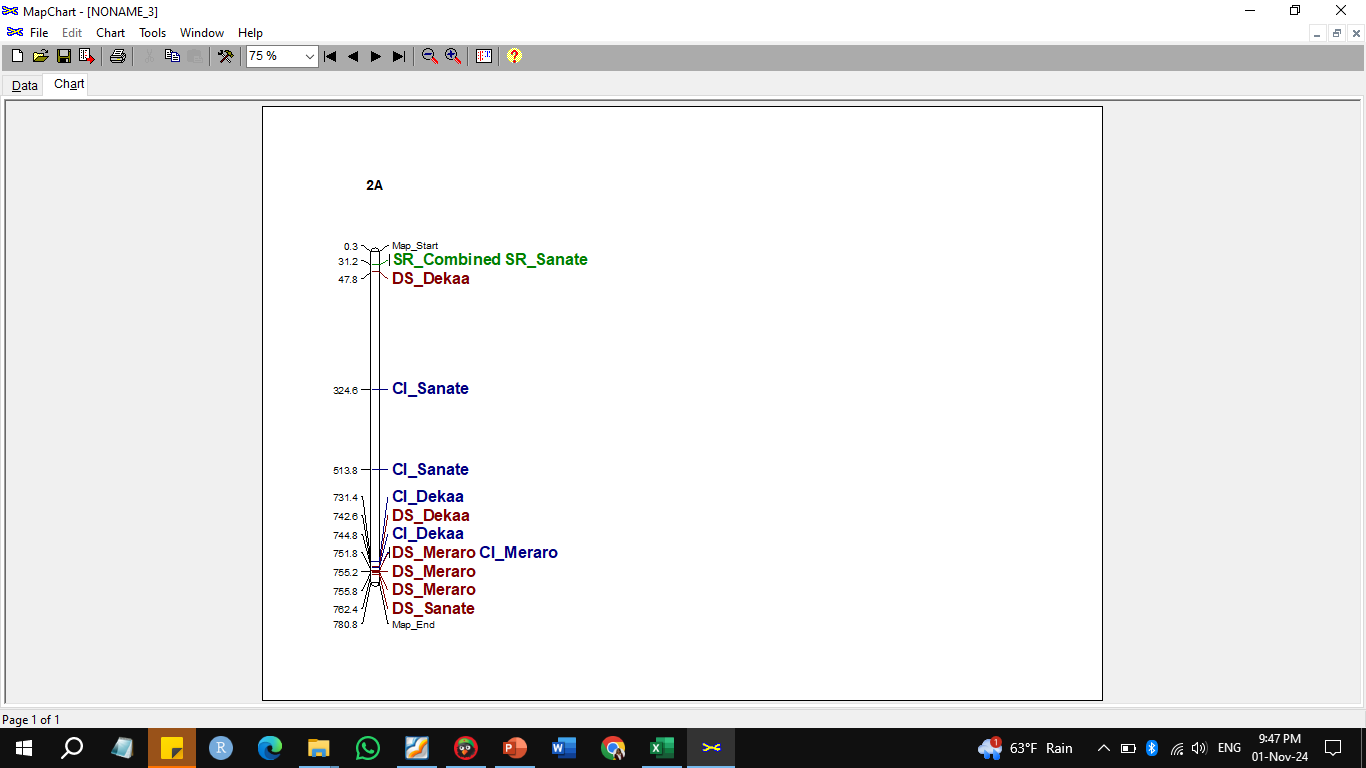


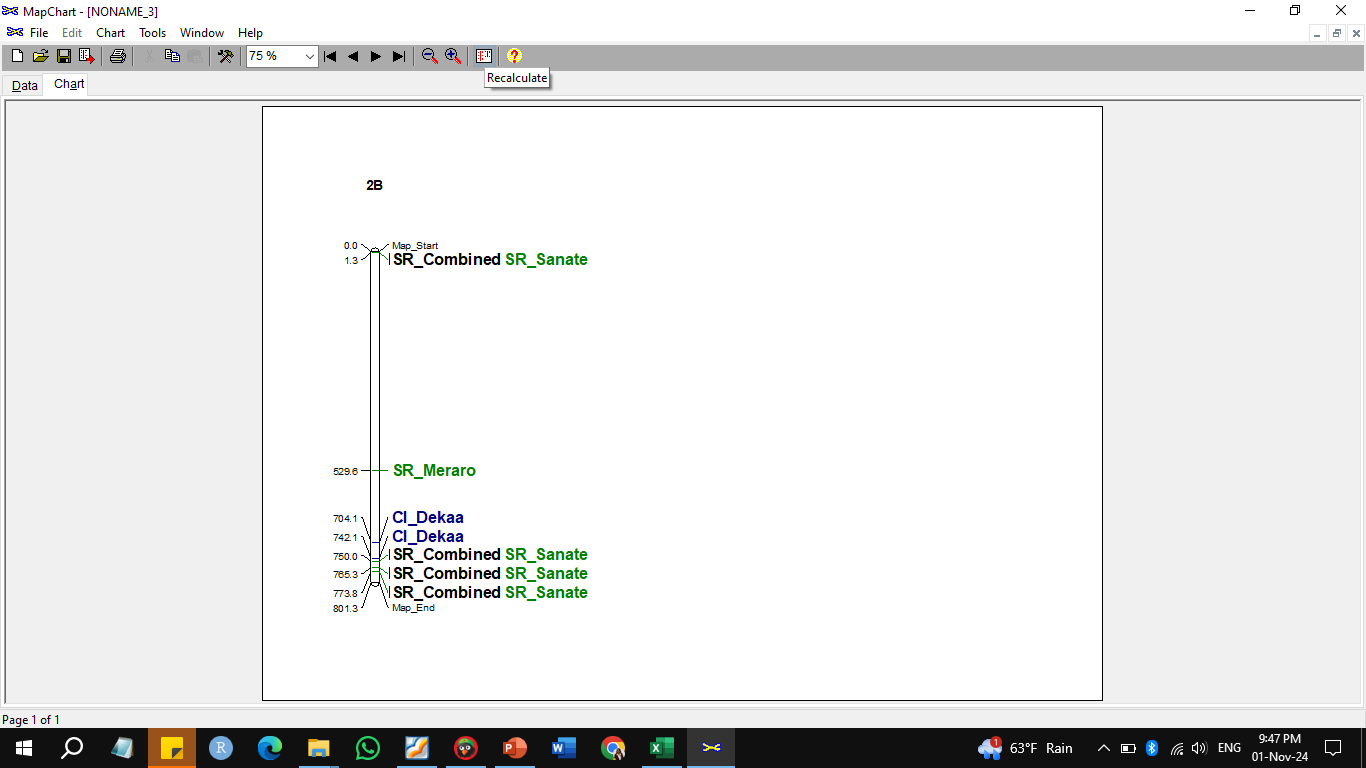

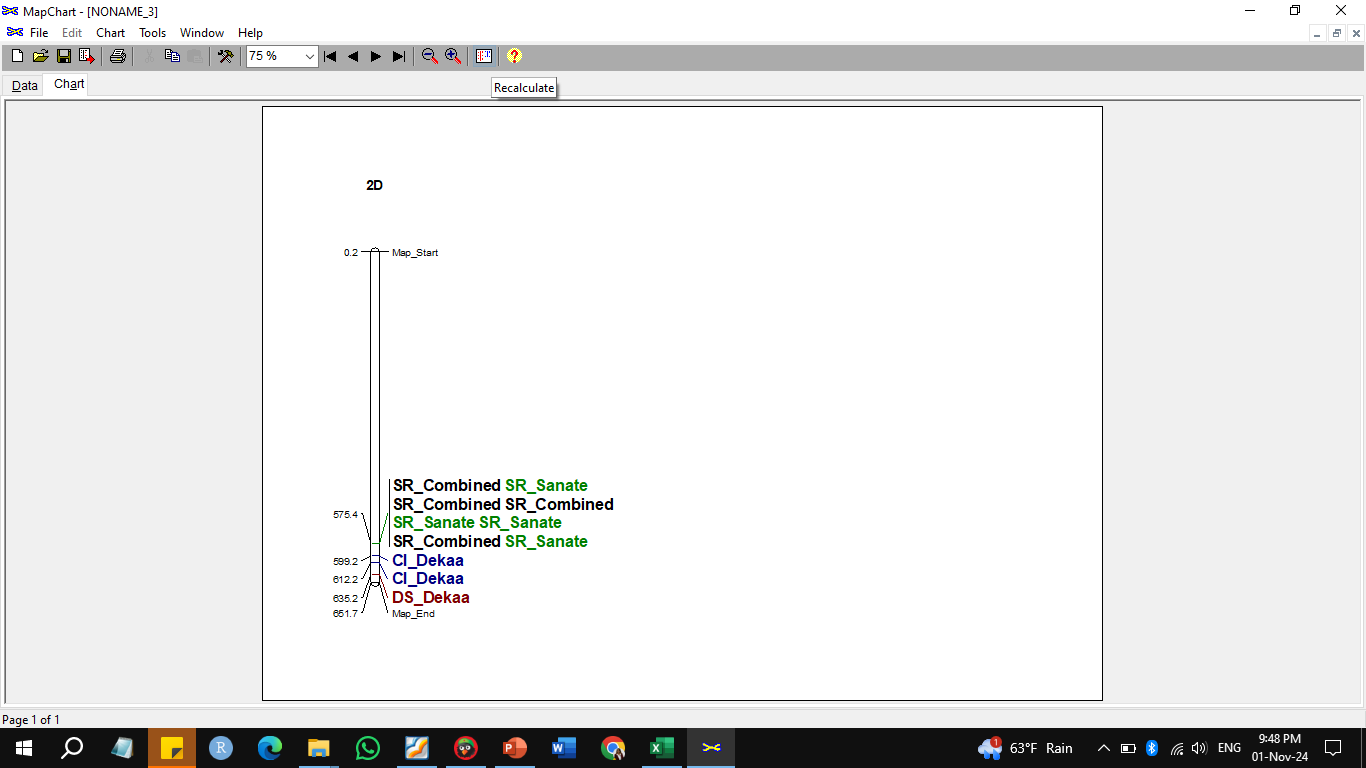

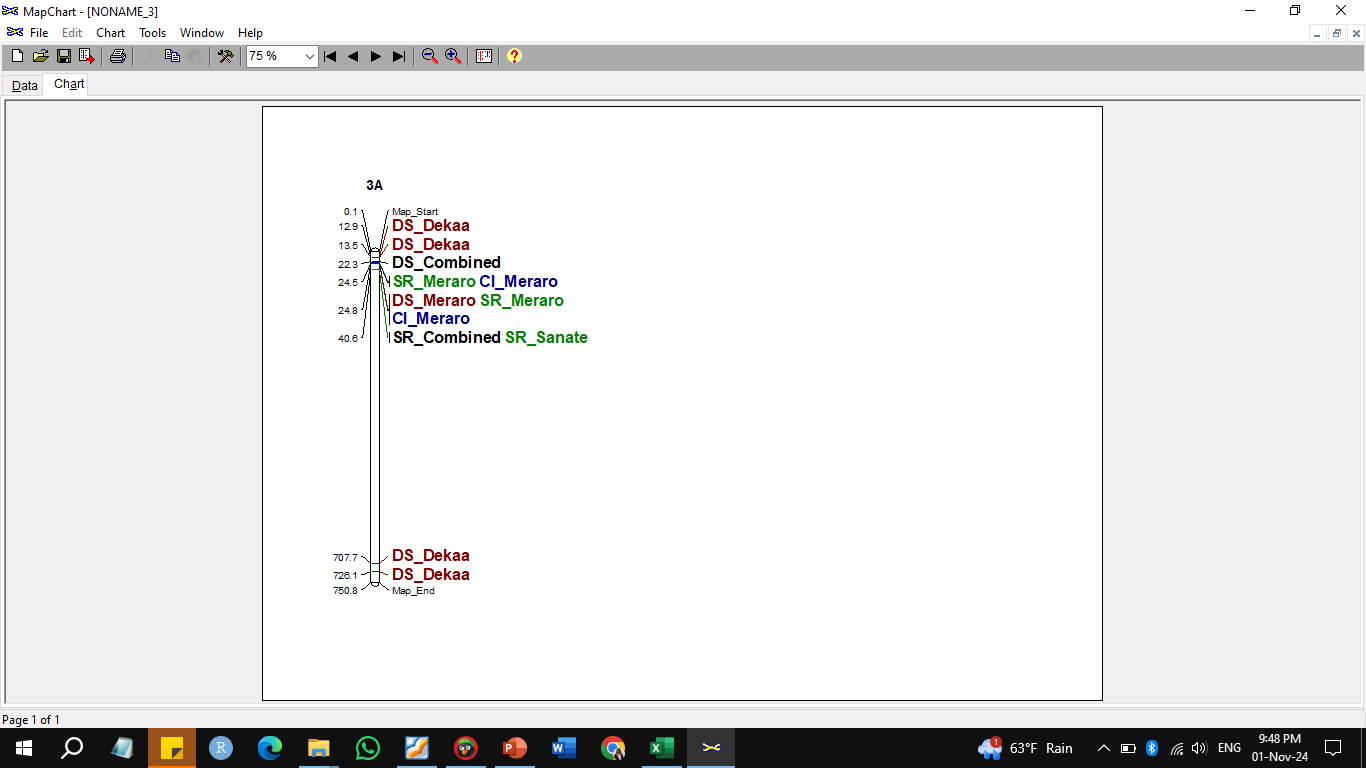


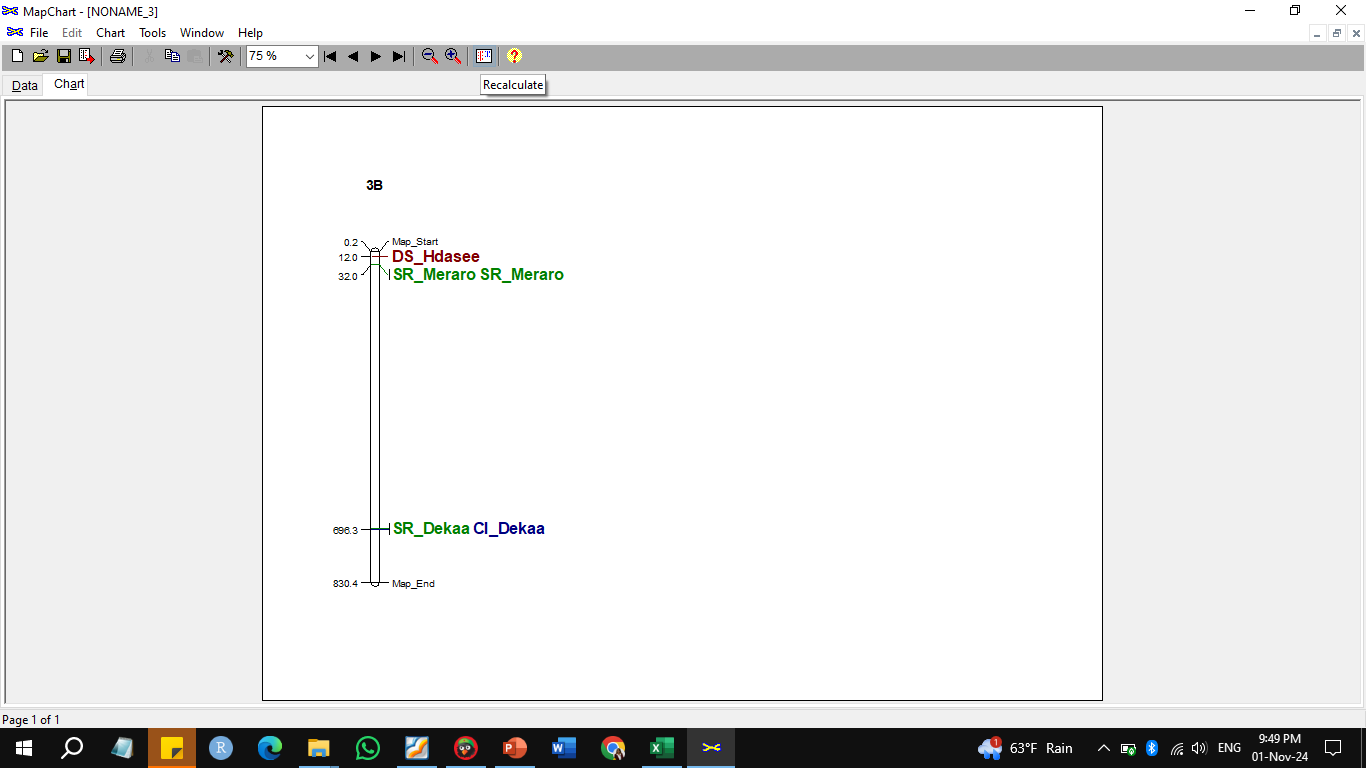

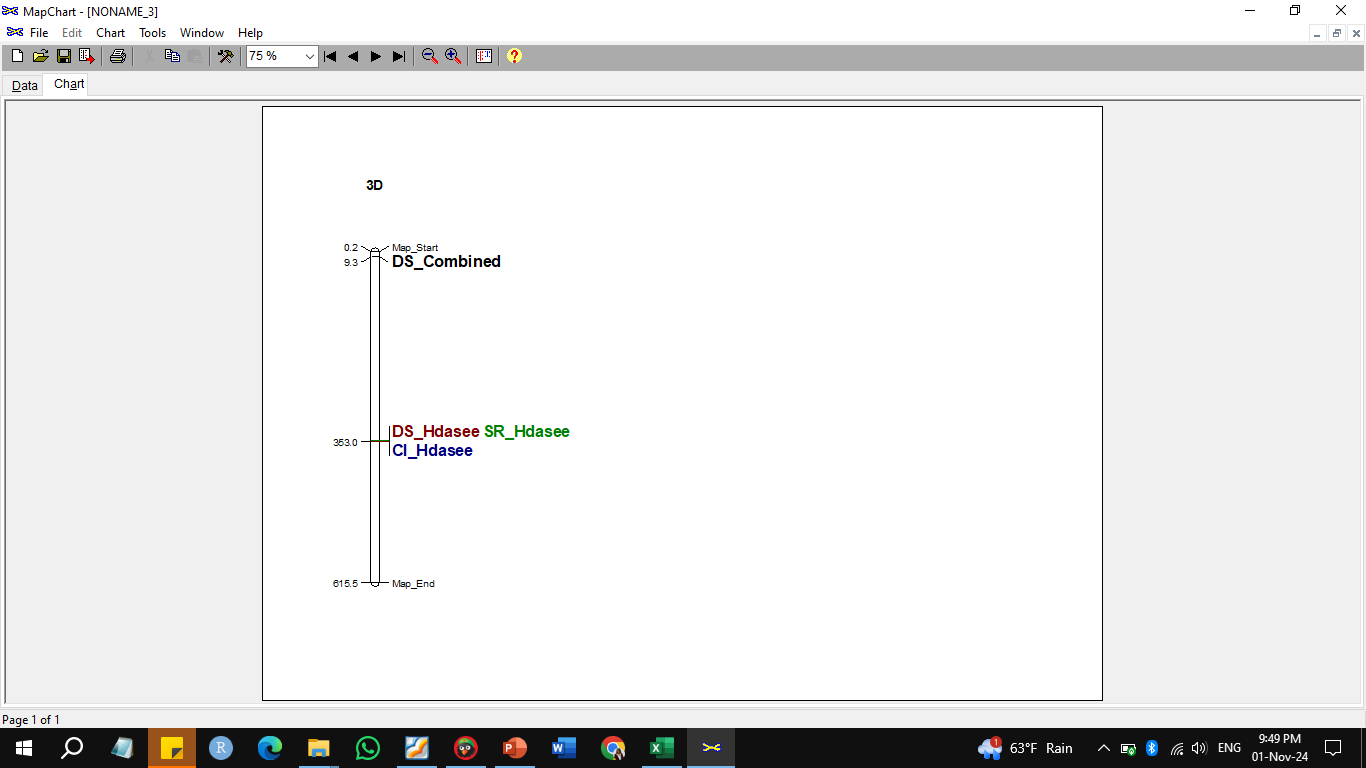

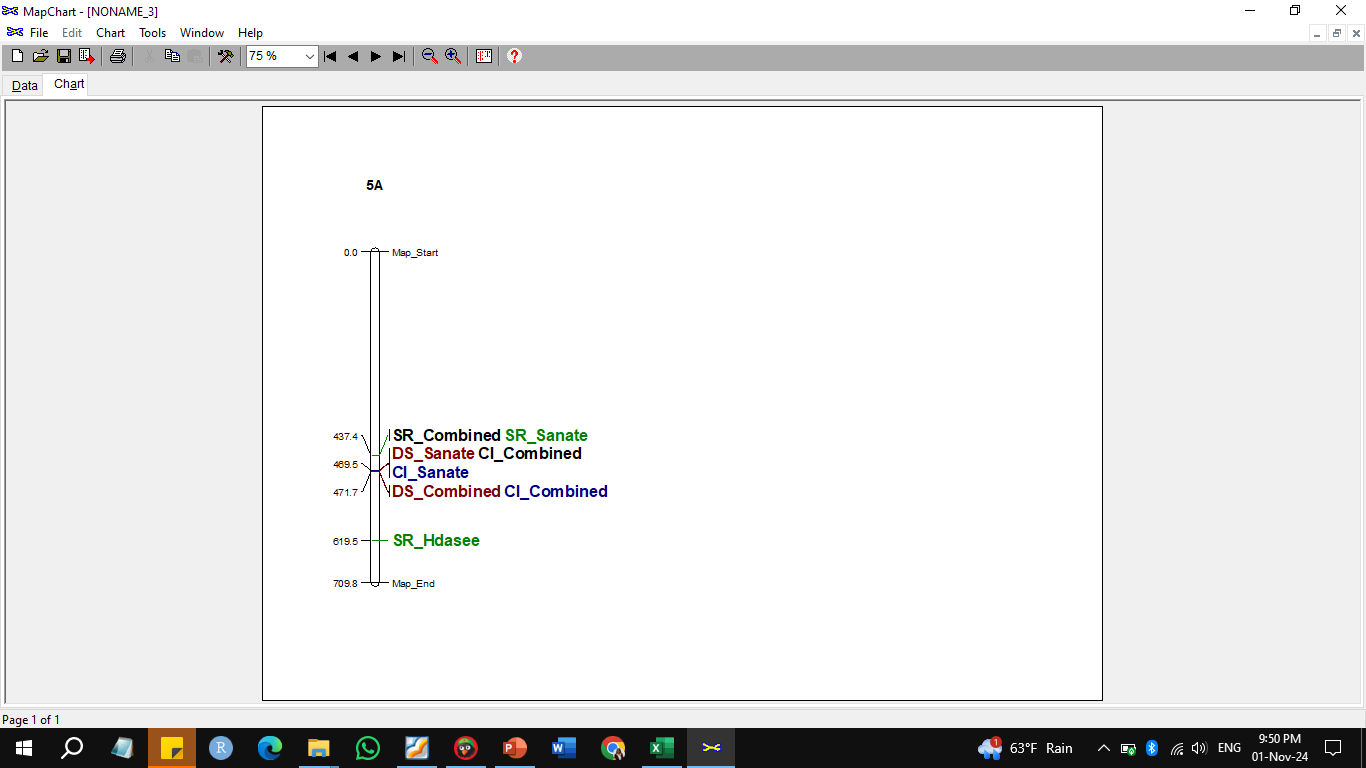


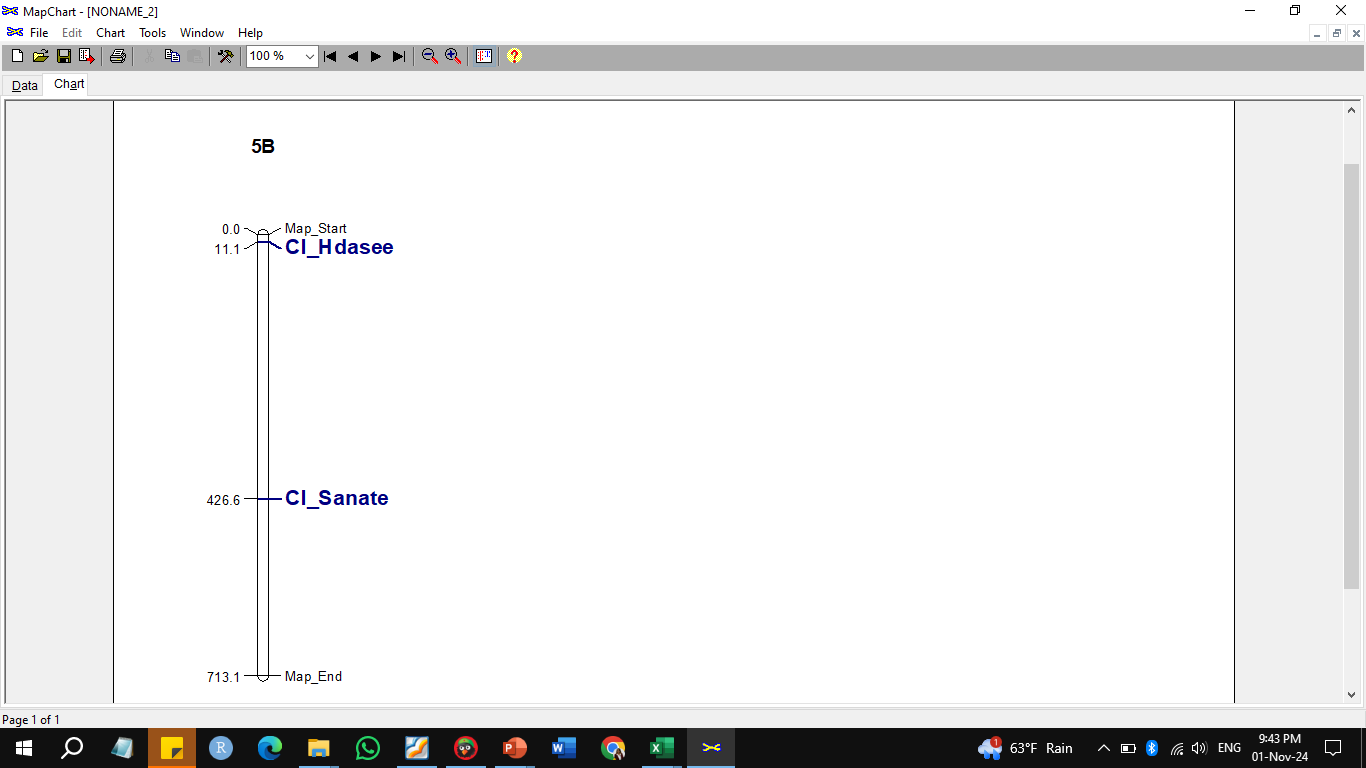

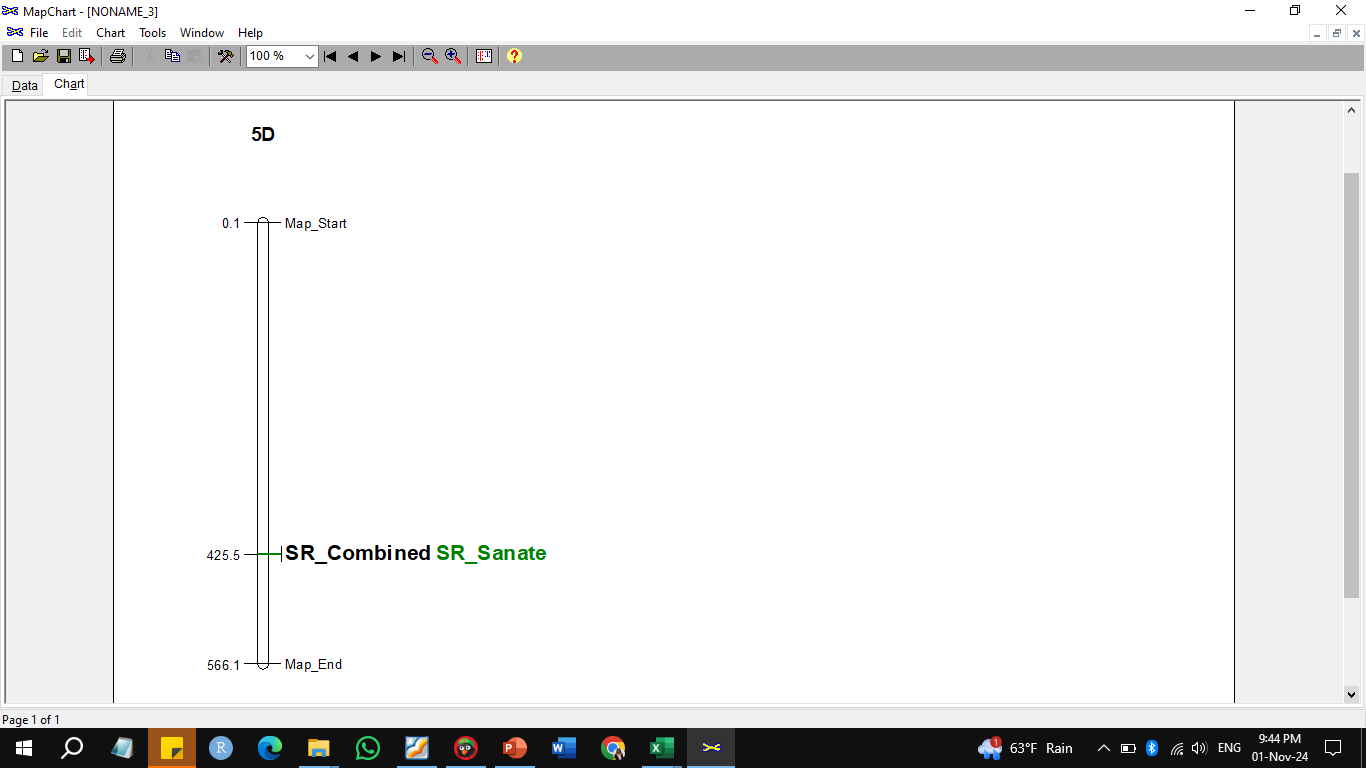

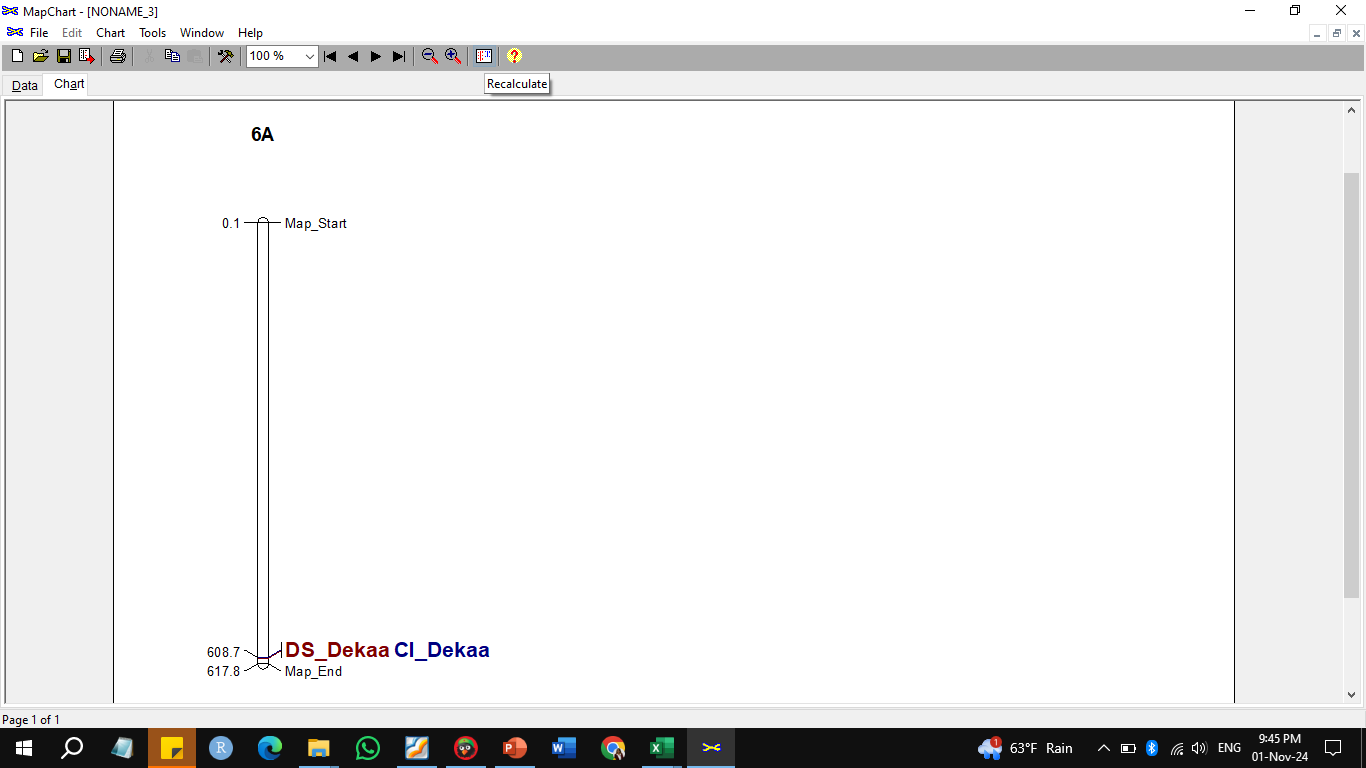


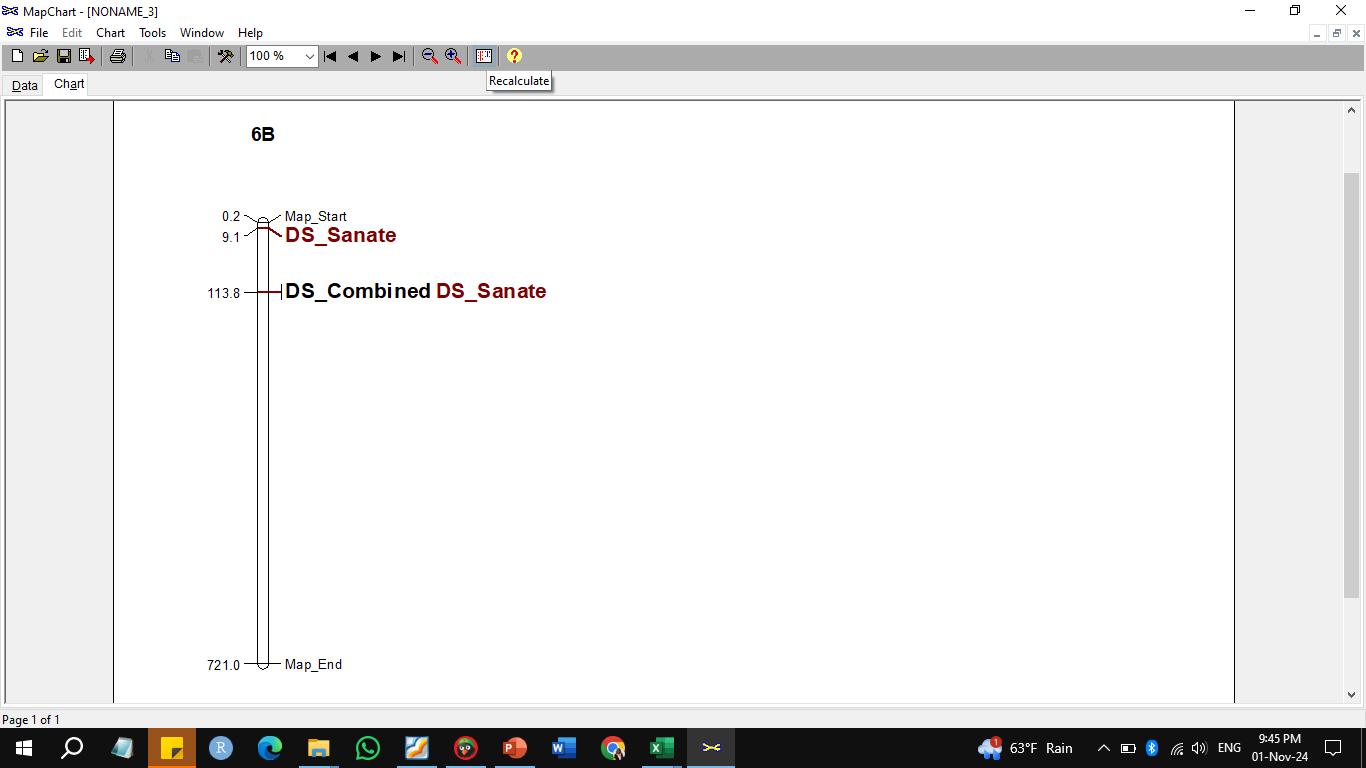

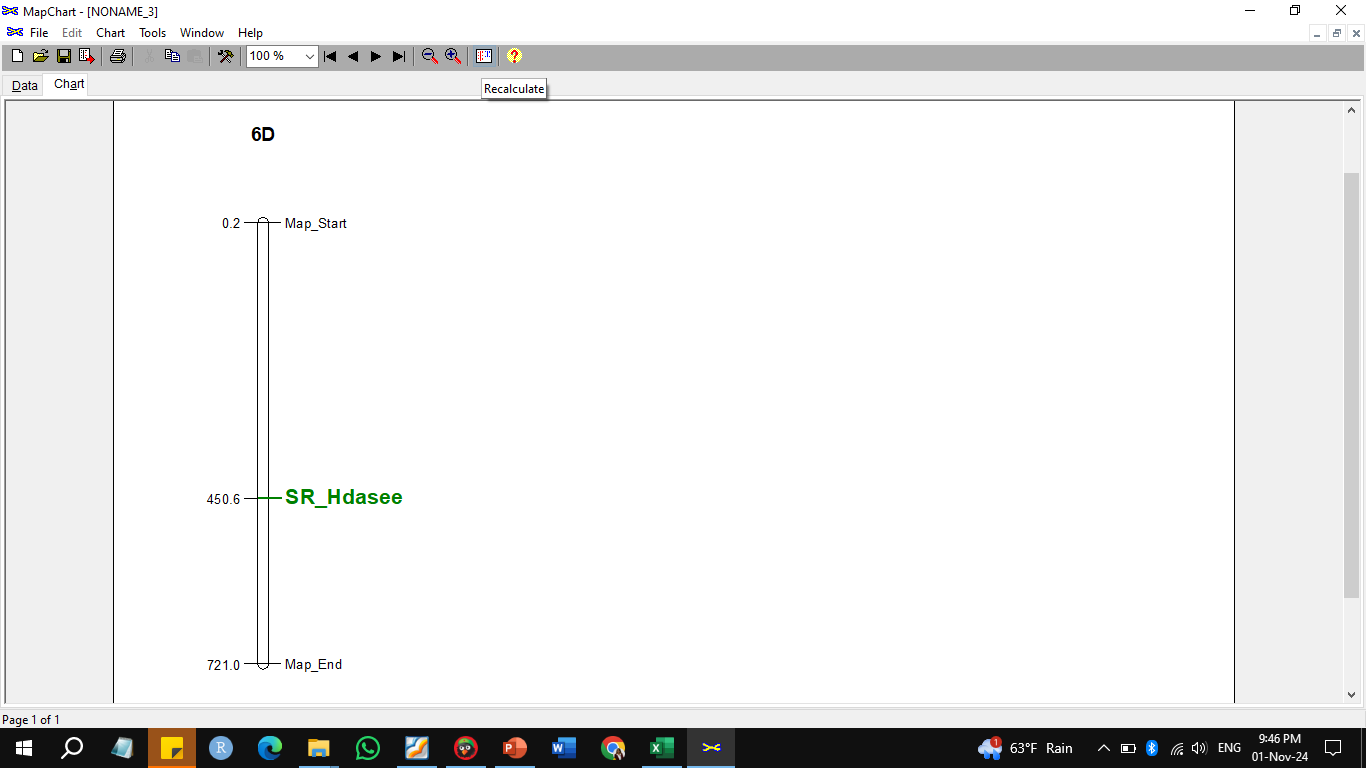

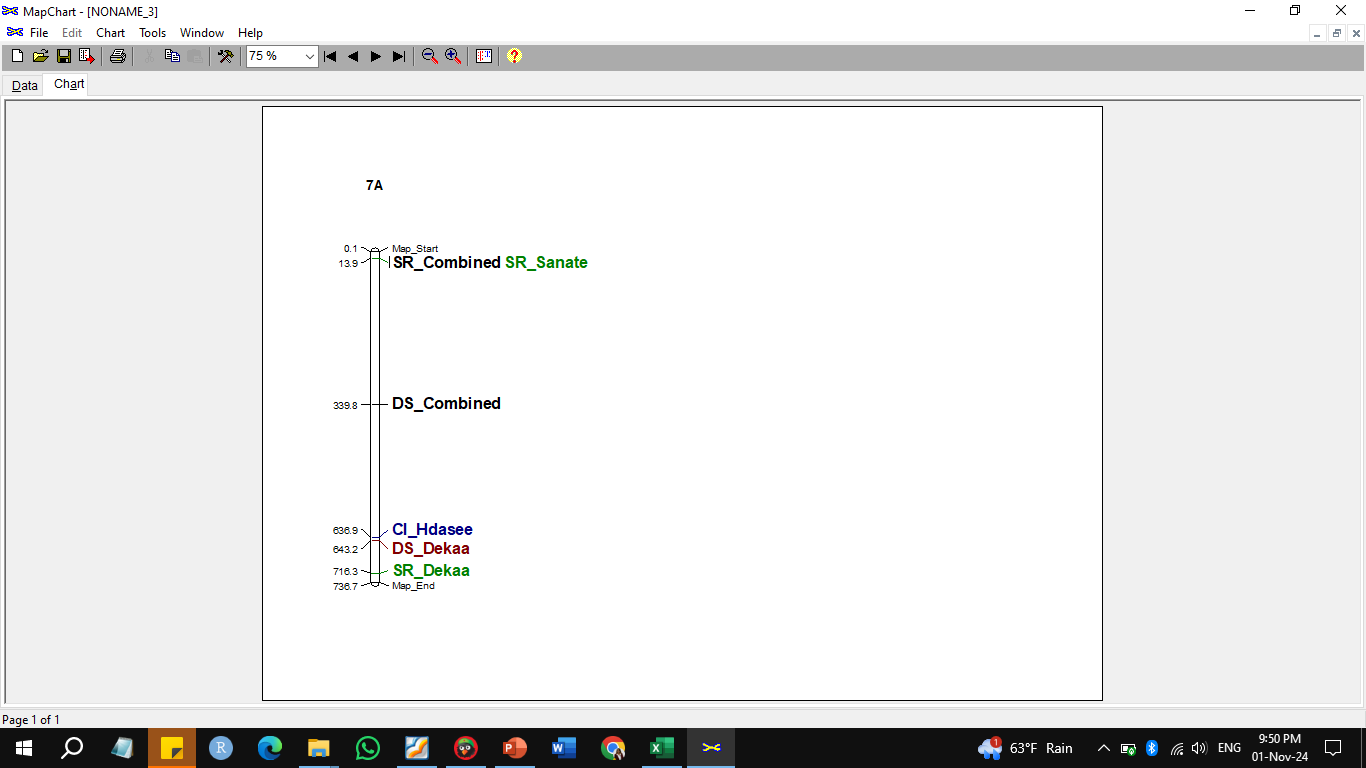


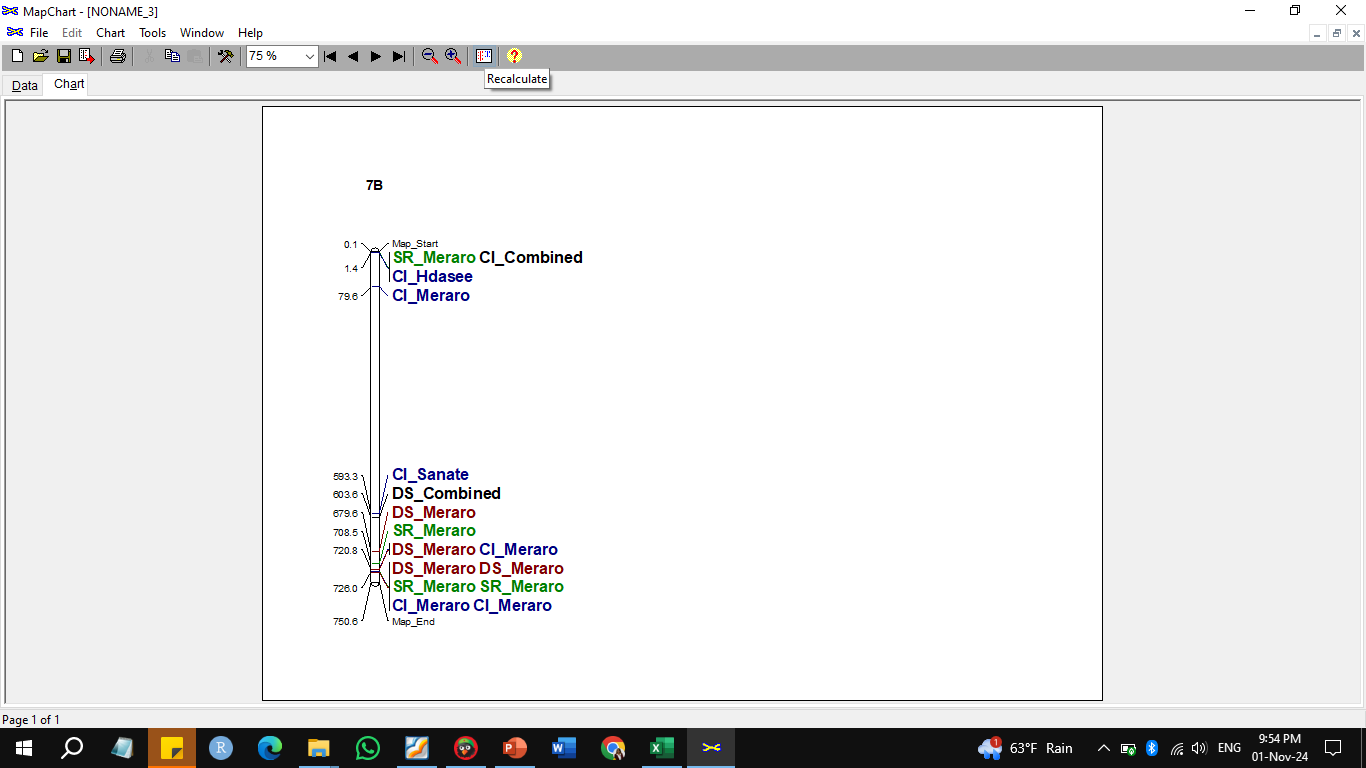

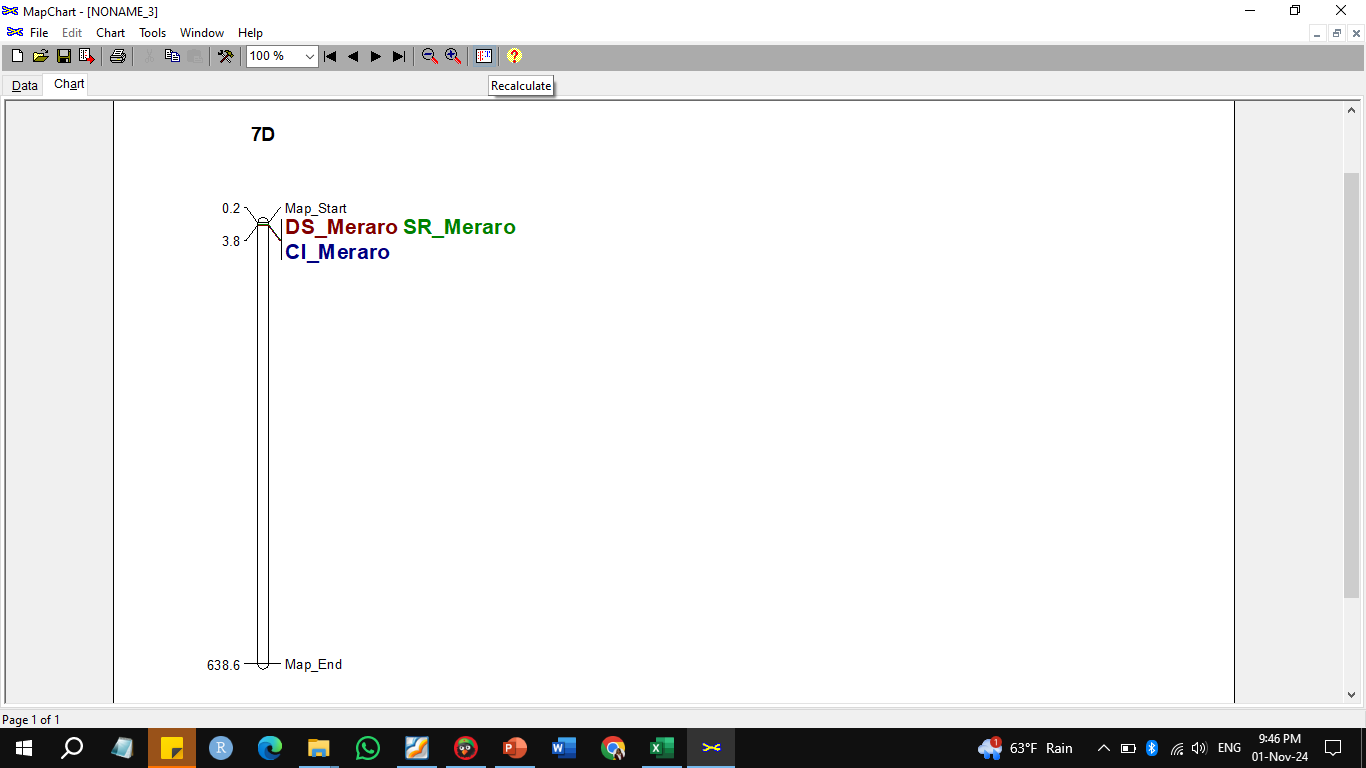


**Supplementary figure 2**. Genomic positions of detected putative QTLs effective for yellow rust resistance. Significant DArTSeq SNPs are presented according to their physical positions on chromosomes in millions base pairs. The putative QTLs identified in this study for the MTAs are indicated on the right sides of the bars. DS_DekaaI = Disease severity for Dekaa isolate, DS_HdaseeI = Disease severity for Hdasee isolate, DS_MeraroI = Disease severity for Meraro isolate, DS_SanateI = Disease severity for Sanate isolate, DS_Combined = Disease severity Combined. SR_DekaaI = Seedling response for Dekaa isolate, SR_HdaseeI = Seedling response for Hdasee isolate, SR_MeraroI = Seedling response for Meraro isolate, SR_SanateI = Seedling response for Sanate isolate, SR_Combined = Seedling response Combined, CI_DekaaI = Confident of infection for Dekaa isolate, CI_HdaseeI = Confident of infection for Hdasee isolate, CI_MeraroI = Confident of infection for Meraro isolate, CI_SanateI = Confident of infection for Sanate isolate, CI_Combined = Confident of infection Combined.
